# Past climatic refugia and landscape resistance explain spatial genetic structure in Oriental beech in the South Caucasus

**DOI:** 10.1002/ece3.9320

**Published:** 2022-09-20

**Authors:** Katarzyna Sękiewicz, Irina Danelia, Vahid Farzaliyev, Hamid Gholizadeh, Grzegorz Iszkuło, Alireza Naqinezhad, Elias Ramezani, Peter A. Thomas, Dominik Tomaszewski, Łukasz Walas, Monika Dering

**Affiliations:** ^1^ Institute of Dendrology Polish Academy of Sciences Kórnik Poland; ^2^ Faculty of Agricultural Science and Biosystems Engineering Georgian Technical University Tbilisi Georgia; ^3^ National Botanical Garden of Georgia Tbilisi Georgia; ^4^ Forest Development Service Ministry of Ecology and Natural Resources of Azerbaijan Baku Azerbaijan; ^5^ Department of Plant Biology, Faculty of Basic Sciences University of Mazandaran Babolsar Iran; ^6^ Faculty of Biological Sciences University of Zielona Góra Zielona Góra Poland; ^7^ Department of Forestry, Faculty of Natural Resources Urmia University Urmia Iran; ^8^ School of Life Sciences Keele University Staffordshire UK; ^9^ Faculty of Forestry and Wood Technology Poznań University of Life Sciences Poznań Poland

**Keywords:** conservation genetics, *Fagus orientalis*, genetic structure, habitat stability, landscape genetics, species distribution modeling

## Abstract

Predicting species‐level effects of climatic changes requires unraveling the factors affecting the spatial genetic composition. However, disentangling the relative contribution of historical and contemporary drivers is challenging. By applying landscape genetics and species distribution modeling, we investigated processes that shaped the neutral genetic structure of Oriental beech (*Fagus orientalis*), aiming to assess the potential risks involved due to possible future distribution changes in the species. Using nuclear microsatellites, we analyze 32 natural populations from the Georgia and Azerbaijan (South Caucasus). We found that the species colonization history is the most important driver of the genetic pattern. The detected west–east gradient of genetic differentiation corresponds strictly to the Colchis and Hyrcanian glacial refugia. A significant signal of associations to environmental variables suggests that the distinct genetic composition of the Azerbaijan and Hyrcanian stands might also be structured by the local climate. Oriental beech retains an overall high diversity; however, in the context of projected habitat loss, its genetic resources might be greatly impoverished. The most affected are the Azerbaijan and Hyrcanian populations, for which the detected genetic impoverishment may enhance their vulnerability to environmental change. Given the adaptive potential of range‐edge populations, the loss of these populations may ultimately affect the specie's adaptation, and thus the stability and resilience of forest ecosystems in the Caucasus ecoregion. Our study is the first approximation of the potential risks involved, inducing far‐reaching conclusions about the need of maintaining the genetic resources of Oriental beech for a species' capacity to cope with environmental change.

## Graphical abstract

We applied landscape genetics and ecological niche modeling, aiming at disentangling the historical and contemporary processes underlying the neutral genetic structure of Oriental beech across the South Caucasus. Our study is the first approximation of the potential risks involved, inducing far‐reaching conclusions about the need of maintaining the genetic resources of Oriental beech for a species' capacity to cope with environmental change.

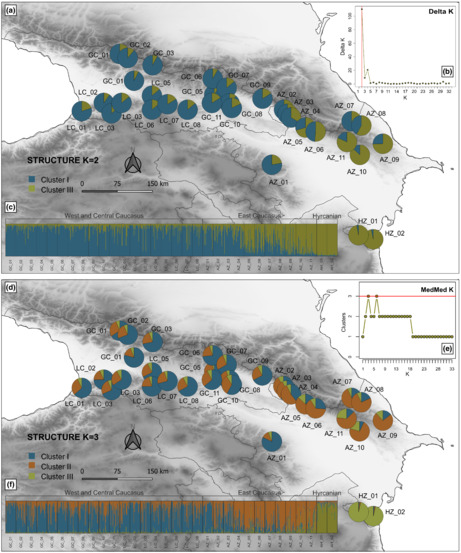

## 1 INTRODUCTION

Trees are known to be playing a substantial role in mitigating the effects of climate change (Anderegg et al., 2020; Bastin et al., 2019). Yet, their long‐term resilience and adaptability is dependent upon genetic diversity, which is currently threatened by climate change itself and by anthropogenic losses of trees on a global scale (Alberto et al., 2013; Hoban et al., 2020; Pauls et al., 2013). Maintaining a high level of genetic diversity and connectivity across the landscape should be a conservation priority, particularly in the world's biodiversity hotspots (Bastin et al., 2019; Fady et al., 2016; Trew & Maclean, 2021).

**FIGURE 1:**
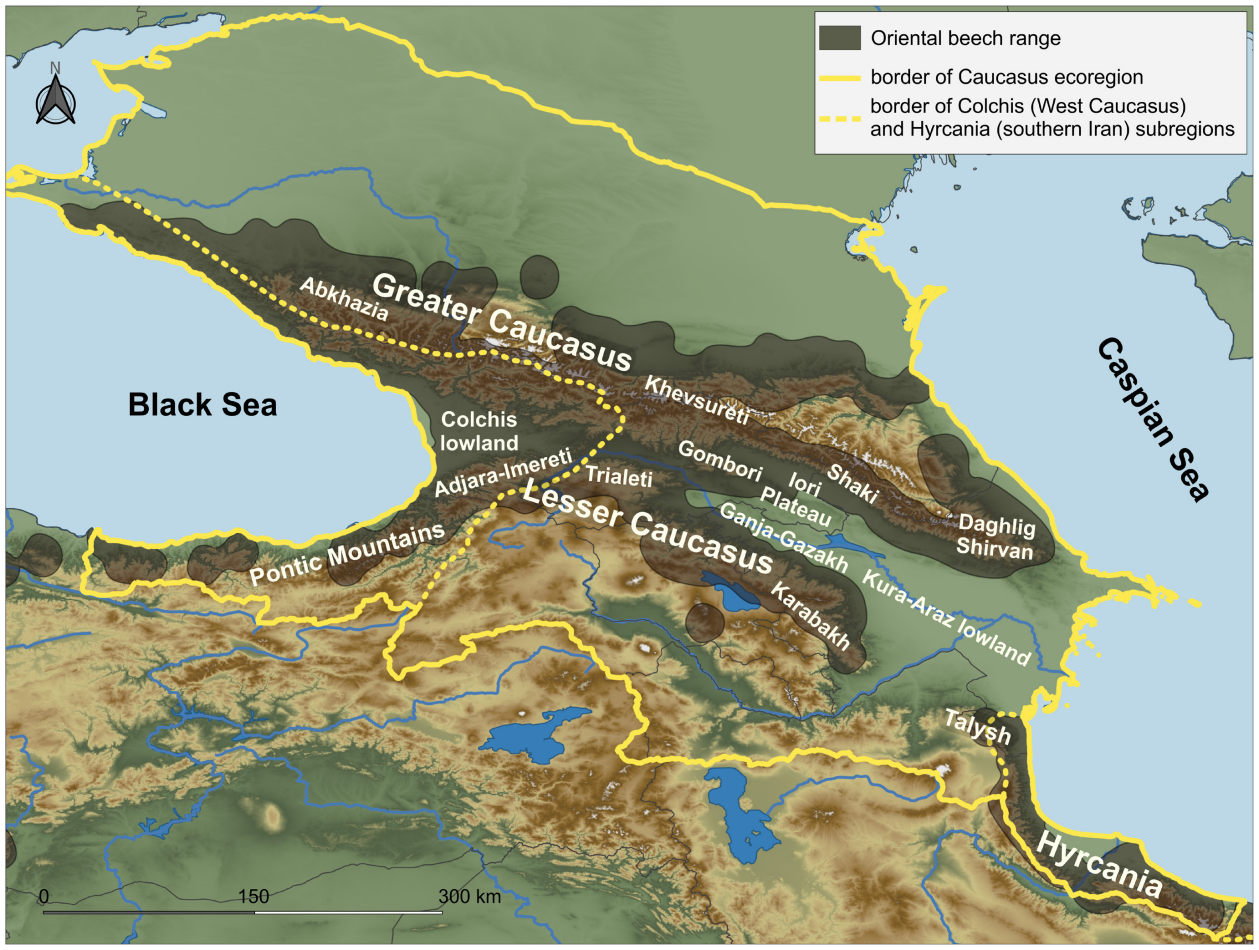
Distribution range of Oriental beech and the major regions of the Caucasus ecoregion.

The Caucasus ecoregion (Figure 1) is one of the biologically richest yet most highly anthropogenically threatened area (Mittermeier et al., 2011; Nikolaishvili & Dvalashvili, 2015; Shatberashvili et al., 2016) and is at high risk of climate change, especially in its eastern part (IPCC, 2022; Nikolaishvili & Dvalashvili, 2015; Shatberashvili et al., 2016). An alarming forest cover loss in the South Caucasus region (i.e., Georgia, Azerbaijan and Armenia) is predicted in this century due to the climatic crisis (Dagtekin et al., 2020; Dering et al., 2021; Zazanashvili et al., 2011), a pattern already observed in Azerbaijan (Buchner et al., 2020). Among the Caucasian broad‐leaved trees, the beech forests are potentially the highly threatened communities – the current distribution of the species may be reduced by over 45% in this century and largely disappear in Azerbaijan and Armenia (Zazanashvili et al., 2011). The most recent projections are far more pessimistic, indicating only limited suitable areas in the North Caucasus and Iran (Dagtekin et al., 2020; Khalatbari Limaki et al., 2021). Additionally, the lower‐elevation populations of this species may be at higher drought risk as was indicated by dendroclimatic analysis (Martin‐Benito et al., 2018). This expected reduction would threaten the stability of the forest in the middle mountain belt, where beech dominates, leading to a pronounced biodiversity loss in the region. In this context, the recognition of factors that governed climate‐driven range shifts of species is needed to assess the vulnerability to future climate threats (Manel & Holderegger, 2013).

Understanding the patterns of microevolutionary responses of tree species to climate changes remains challenging due to the complex factors involved in the process. These include the uncertainty of future climate scenarios, limitations of species distribution projections, and doubts related to the adaptive potential or the spatio‐temporal environmental heterogeneity across species ranges (Alberto et al., 2013; Capblancq, Fitzpatrick, et al., 2020). Nevertheless, recognition of spatial variation in genetic composition can give insights not only into the impact of the past climate on species' biogeography but also on current population dynamics, particularly the possible genetic consequences of range shifts and, in long term, understanding the effects of climate change (Gavin et al., 2014; Hoffmann et al., 2015). Genetic variation across the range of a species is determined by the interplay of demographic, ecological, and evolutionary processes (Manel & Holderegger, 2013; Orsini et al., 2013). Several theoretical patterns can affect the distribution of genetic diversity, including isolation by distance (IBD), isolation by environment (IBE), isolation by resistance (IBR), or isolation by colonization (IBC; Orsini et al., 2013). However, disentangling the relative contribution of geographic, historical, and contemporary landscape factors affecting these patterns is challenging because they are often spatially correlated, leading to overlapping effects (Nadeau et al., 2016; Orsini et al., 2013). Unraveling the processes underlying the spatial genetic patterns and quantifying the importance of environmental variables in structuring population genetic variation is crucial to managing species and ensuring their long‐term sustainability in a changing environment (Hoffmann et al., 2015; Manel & Holderegger, 2013; Orsini et al., 2013). This is especially important for species occurring in heterogeneous mountainous environments, which are particularly sensitive to the impacts of climate change (Beniston, 2003).

To test for factors shaping spatial variation in neutral genetic composition in a highly heterogeneous landscape, we focused on Oriental beech (*Fagus orientalis* Lipsky), the most ecologically and economically important tree in the Caucasus (Tarkhnishvili, 2014; Zazanashvili et al., 2011). Its current distribution (Figure 1) includes the Northern Anatolian Mts., the Caucasus Mts., the Talysh Mts. (southeastern Azerbaijan), and the Hyrcanian forests (northern Iran), with isolated populations found in the Amanos and Taurus Mts. (southern Turkey; Browicz & Zieliński, 1982). Studies suggest that during the Last Glacial Maximum (LGM), the Pontic Mts. (Turkey), the Colchis (western Caucasus), and the Hyrcanian (Iran) regions were the main refugia for forest trees, including Oriental beech (Connor & Kvavadze, 2009; Dagtekin et al., 2020; Leroy & Arpe, 2007; E. Ramezani et al., personal communication; Shatilova et al., 2011). While the postglacial migration of the Caucasian temperate forest mostly relied on the Colchis refugium (Connor & Kvavadze, 2009; Tarkhnishvili et al., 2012), the Hyrcanian area is perceived more as the sanctuary of the Neogene flora with limited input into the re‐colonization (Akhani et al., 2010). Growing evidence highlights the asymmetrical contribution of the Colchis and Hyrcanian refugia in shaping the modern patterns of genetic structure, suggesting west–east postglacial expansions in the Caucasus and the predominant role of the Colchis (Dering et al., 2021; Parvizi et al., 2019; Tarkhnishvili, 2014). The other detected pattern of interspecific divergence in the Caucasus reflects the vicariance process in these isolated glacial refugia (Christe et al., 2014; Maharramova et al., 2018). Therefore, the Caucasus ecoregion offers an excellent abiotic template to investigate the effects of multiple landscape factors on the contemporary genetic structure of Oriental beech.

We focus on conceptual frameworks that point out the interplay of the neutral and adaptive processes in structuring the neutral genetic diversity in species, as proposed by Orsini et al. (2013). However, due to methodological constraints related to using neutral markers, we mainly discuss neutral processes with some indirect hint about adaptive divergence. Based on available studies (Connor, 2006; Dagtekin et al., 2020; Dering et al., 2021; Tarkhnishvili, 2014), we assumed that the current genetic patterns in Oriental beech have mostly been governed by the colonization history but modified by environmental and adaptive processes. Therefore, we expected decreasing genetic diversity away from the main refugial area due to repeated founding events along the migration routes (Hampe & Petit, 2005). On the contrary, the complex landscape of the Caucasus could induce adaptation to specific habitats promoting intraspecific divergence resulting in a detection of the IBE pattern (Orsini et al., 2013). The adaptive processes may also interact with the neutral ones resulting in the IBC pattern when the local adaptation reinforces the founder effects during range expansions and drive IBC under a monopolization scenario. In this case, the founder effect leads to considerable genetic differentiation among populations and no clear link between the genetic pattern and the spatial and environmental gradients (Orsini et al., 2013). In addition, the long persistence of the species in the isolated refugia could drive a Colchic‐Hyrcanian genetic split among populations in those subregions. However, given the species' high potential for gene flow, we may expect a partial eroding of the genetic signal left by the historical factors, leading to overall moderate differentiation. Assessing the future persistence of Oriental beech populations in the Caucasus requires understanding which extrinsic factors determined the current patterns of genetic diversity and connectivity while accounting for their complex evolutionary history.

Here, we applied landscape genetics and ecological niche modeling, aiming at disentangling the historical and contemporary processes underlying the neutral genetic structure of Oriental beech across the South Caucasus. Specifically, we address the following questions: (1) Is genetic diversity spatially structured across the landscape, (2) If yes, what historical, environmental, or spatial processes drive detected patterns of genetic diversity and differentiation? and (3) What are the potential risks involved due to possible changes in the species distribution under future climate projections? By understanding how the species' genetic structure is associated with current climate variables, we can make the first approximations about the potential risks involved under a future climate (Manel & Holderegger, 2013). Finally, we discuss the implications of the results for the conservation and management of Oriental beech, a keystone tree species of forest ecosystems in the Caucasus.

## 2 MATERIALS AND METHODS

### 2.1 Population sampling and genotype acquisition

**FIGURE 2:**
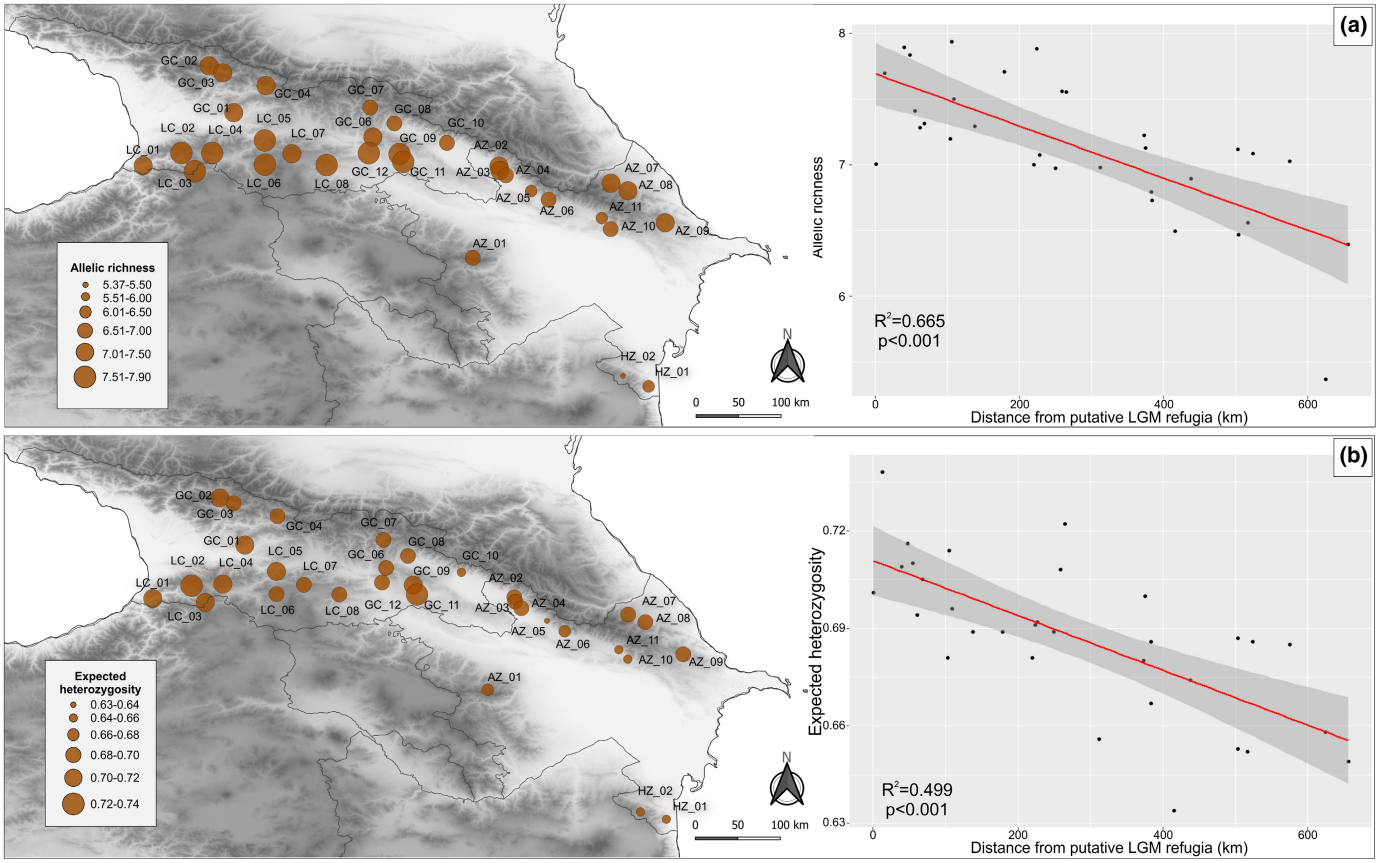
Locations of the sampled populations of Oriental beech in Georgia (GC, Greater Caucasus; LC, Lesser Caucasus) and Azerbaijan (AZ, East Caucasus; HZ, Hyrcania). Spatial distribution of genetic diversity across the landscape (left panel) with the relationship between both genetic parameters and distance from putative LGM refugial area (Dist_LGM_; right panel) – a represents allelic richness (*A*_R_) and b expected heterozygosity (*H*_E_). The population abbreviations as in Table 1; Appendix S1: Table S1.1.

Sampling covered 32 natural populations of Oriental beech (857 individuals) collected over the entire species range in the South Caucasus (Figure 2; see Appendix S1: Table S1.1; Appendix S2: Figure S2.1). Specifically, 19 populations were sampled in the Greater (GC) and Lesser (LC) Caucasus, 11 populations in the Azerbaijan part of the Eastern Greater Caucasus (AZ), and two populations in the Talysh Mts. (southeastern Azerbaijan), which represents the Hyrcanian forests (HZ).

Genomic DNA was extracted from the leaf according to the CTAB protocol (Dumolin et al., 1995). Individuals were genotyped using 13 nuclear microsatellite loci (nSSRs) originally developed for *Fagus sylvatica* (Pastorelli et al., 2003; Pluess & Määttänen, 2013; Appendix S3: Table S3.2). Details on PCR reaction, fragment separation, and genotyping are described in Appendix S1.

### 2.2 Population genetic analyses

#### 2.2.1 Diversity and differentiation

GENEPOP v 4.7 (Raymond & Rousset, 1995) was used to check for the departures from the Hardy–Weinberg equilibrium and linkage disequilibrium (LD). To test for the significance of LD, we used Fisher's exact test with the Bonferroni correction. All loci were checked for the presence of null alleles using INEST v.2.2 according to the individual inbreeding model (IIM; Chybicki & Burczyk, 2009).

To assess how diversity varied within and among populations, we calculated the mean number of alleles (*A*), observed (*H*_O_), and expected (*H*_E_) heterozygosity using INEST whereas the number of private alleles (PA) was computed using GenAlEx (Peakall & Smouse, 2012). The allelic richness (*A*_R_) based on the rarefaction method was obtained using FSTAT v.2.9.3 (Goudet, 1995). A comparison of genetic diversity parameters (*A*_R_, *H*_E_, and *F*_IS_) among the main population demes (Lesser Caucasus, Greater Caucasus, Azerbaijan, and Hyrcania) was tested in FSTAT with 10^4^ permutations.

To estimate the inbreeding coefficient including “null alleles” correction (*F*_IS_Null), we used the individual inbreeding model (IIM) implemented in INEST. The calculations were run with 5 × 10^5^ MCMC iterations with every 200th updated and a burn‐in of 5 × 10^4^. To assess the factors affecting the homozygosity level in populations, the competition of the full model (“nfb” = null alleles, inbreeding coefficient, and genotyping failures, *F*_IS_ > 0) with the random model (“nb” = null alleles, genotyping failures, *F*_IS_ = 0) was applied based on the Deviance Information Criterion (DIC).

The overall population genetic differentiation was estimated using the Wright's fixation index (*F*_ST_) with the Excluding Null Alleles (ENA) correction implemented in FreeNA (Chapuis & Estoup, 2007). The confidence interval for *F*_ST_ was determined using bootstrap resampling over loci method with 10,000 replications. To measure the extent of differentiation within the regions, and among the populations, the pairwise *F*_ST_ following Weir and Cockerham was calculated with ENA correction.

The M‐ratio method (Garza & Williamson, 2001) implemented in INEST was used to detect the signature of a recent bottleneck. The M‐ratio (MR) was estimated by simulating analysis with 10^5^ coalescent replicates under the two‐phase mutation model (TPM) assuming a proportion of one‐step mutations (ps) of 0.22 and a mean size of multi‐step mutations (Δg) of 3.1. The significance of the deficiency in the M‐ratio was tested using the Wilcoxon signed‐rank test.

#### 2.2.2 Range‐wide population structure

To define the population's genetic structure and admixture, we used STRUCTURE v.2.3 (Pritchard et al., 2000). We assumed admixture models and correlated allele frequencies without prior information on population memberships. Ten replicate runs of independent subsampling were performed for each genetic cluster (*K*), ranging from one to 33 with a burn‐in period of 10^5^ steps followed by 2 × 10^5^ MCMC iterations. Following Cullingham et al. (2020), we applied different *K*‐selection methods, including the log probability of the data (Ln Pr (*X*|*K*); Pritchard et al., 2000), Evannos' Δ*K* (Evanno et al., 2005) and the algorithm based on the mean or median membership coefficient (*Q*) (Puechmaille, 2016). To obtain the *K*‐selection plots, we used StructureSelector (Li & Liu, 2018) while CLUMPAK (Kopelman et al., 2015) was used to summarize and visualize the replicate runs. According to Puechmaille (2016), we considered clustering results in which a mean membership coefficient (*Q*) given to genetic clusters is >0.5 to exclude the spurious cluster, which constitutes a doubtful biological grouping. However, being aware of the complexity of the *K*‐selection procedures, we included all clustering results that warrant biogeographic interpretation (Cullingham et al., 2020).

#### 2.2.3 Species distribution modeling

We used species distribution models (SDMs) to calculate the three landscape metrics: current and past climatic suitability, and distance from the climatically stable area in the LGM. As our point was also to predict the possible changes in the species distribution in future, we constructed the theoretical distribution of the species under different future climate scenarios.

The species occurrence acquisition is detailed in Appendix S2, and for the final modeling procedure, the occurrences dataset hosted 810 unique records (see Appendix S2: Figure S2.1).

The maximum entropy approach implemented in MaxEnt 3.4.1 (Phillips et al., 2004) was applied to build the models. To construct the model of the species' potential distribution for current condition and for future projections (2061–2080), a set of 19 bioclimatic variables at 30 arc‐sec resolution were retrieved from CHELSA 1.2 (Karger et al., 2017). Further, these data were upscaled to match the resolution and extent of the bioclimatic variables in QGIS. We used the variance inflation factor (VIF) to eliminate predictor collinearity using the *vif* function implemented in the *usdm* R package (Naimi et al., 2014). Variables with large VIF values (>5) were excluded one by one using a stepwise procedure. Finally, the resulting dataset contained nine environmental variables: the annual mean temperature (bio1), isothermality (bio3), temperature seasonality (bio4), mean temperature of the wettest quarter (bio8), mean temperature of the driest quarter (bio9), precipitation seasonality (bio15), precipitation of the warmest quarter (bio18), and the precipitation of the coldest quarter (bio19). The same set of bioclimatic variables at 2.5 arc‐min resolution obtained from PaleoClim (Fordham et al., 2017) was applied for past projection during the Last Glacial Maximum (LGM; ca. 21 ka). For past projection, data were obtained from PaleoClim (Fordham et al., 2017); for current and future conditions from CHELSA 1.2 (Karger et al., 2017). The distribution of the species during the LGM (ca. 21 ka) was projected using the Community Climate System Model (CCSM4; Karger et al., 2017), while future projections (2050–2080) based on the Coupled Model Intercomparison Project Phase 5 (CMIP5) following the Representative Concentration Pathways – RCP 4.5 and RCP 8.5 scenarios (Collins et al., 2013).

Maxent was run with 100 replicates using bootstrap resampling, the maximum number of iterations was set at 10^4^, and the convergence threshold was set at 10^−5^ with the logistic output of the model prediction for suitability. The “random seed” option was applied to validate the models, where 20% of the occurrence points were random sampling as test data, the remaining points were used as training data, and a random test partition was used for each run. Model accuracy was evaluated using the area under the curve (AUC) values of the receiving operator curve (ROC) as a threshold‐independent evaluation metric (Mas et al., 2013). Results of SDMs across the landscape were visualized using QUANTUM GIS 3.24.0 “Tisler” (QGIS.org, 2022), while habitat suitability and average altitude in the theoretical range of the species were calculated in SAGA GIS (Conrad et al., 2015). To illustrate the extent of the future environmental change among major distributional domains of the species (Greater Caucasus, Lesser Caucasus, Azerbaijan, and Hyrcania), we compared the bioclimatic parameters that had the highest contribution to the SDMs and the current and future habitat suitability using bar charts.

To define populations with high priority for conservation based on genetic data, we applied the Reserve Selection algorithm implemented in DIVA‐GIS v.7.5 (Hijmans et al., 2001) using the complementarity site selection procedure. The procedure first identified the population that captures the highest allelic richness across all studied sites; subsequently, it selects an additional location containing the highest richness after excluding the alleles already present in previously selected populations. This analysis efficiently identifies the minimum number of geographical units needed to conserve all intraspecific genetic diversity. The results were visualized in QGIS against the future habitat suitability projected under the RCP8.5. Moreover, to identify the climatic refugia for Oriental beech, we estimated areas of stability (habitat suitability >60%) defined as a region of overlap between the projected future (RCP4.5 and RCP8.5) and current distribution patterns that support the long‐term species occurrence in these regions. For this purpose, the binary Maxent model outputs (30 arc‐sec) for the future projections were aggregated to the potential current distribution (30 arc‐sec) using raster calculator in QGIS. Results of the areas of stability were visualized using QGIS.

#### 2.2.4 Predictors of genetic diversity and gene flow

To detect the drivers governing the spatial distribution of genetic diversity, we employed generalized linear models (GLMs) to test the hypothesis that past climate may explain the observed pattern (Hampe & Petit, 2005; Hewitt, 2000) using the *glm* R function (R Core Team, 2022). The hypothesis emphasizes that higher genetic diversity is related to the proximity of populations to LGM refugia and subsequent decrease due to postglacial migration. In the models, we considered five explanatory variables, including current habitat suitability (HS_CURR_), distance from LGM refugium in Colchis (Dist_LGM_), genetic admixture (*G*_ADMIX_), latitude and longitude, while *A*_R_ and *H*_E_ were used as response variables.

To generate the LGM niche centroid of the species distribution, we used habitat suitability predicted by Maxent, applying the “centroids” option in QGIS. The Euclidean distance was used as a metric of the population distance from the niche centroid. The extent of admixture based on the STRUCTURE result at *K* = 2 (see section 3) was estimated using the “genetic admixture index” obtained according to the procedure described by Ortego et al. (2015). Models were compared using the Nagelkerke pseudo *R*‐squared, Akaike Information Criterion (AIC), and Akaike weights (*w*_i_) calculated using the function *compareGLM* in *rcompanion* R package (Mangiafico, 2022) and *model.sel* in the *MuMIn* R library (Bartoń, 2020).

A series of distance‐based redundancy analyses (dbRDA) were performed to unravel the relative contribution of climate (*clim*.), geography (*geo*.), recent migration (*mig*.), demographic history (*anc*.), and topographic heterogeneity (*top*.) in explaining the detected genetic differentiation (Legendre & Legendre, 2012). To do this, we used the pairwise Slatkin's linearized *F*_ST_ (*F*_ST_/1−*F*_ST_) with ENA correction as the response variable and a set of explanatory/conditioning variables described below. The analyses were run using the function *capscale* in *vegan* R package (Oksanen et al., 2020). First, we tested all the different combinations of all explanatory and conditioning variables in the partial dbRDA to define the “pure” effect of variables and ignore the insignificant variables. Specifically, this constrained ordination approach allowed us to decompose the portion of genetic variance explained by each set of the variables and detect the relative effect of a specific variable by removing the confounding effect of the remaining associated variables that can be spatially correlated (Legendre & Legendre, 2012). Significance of the associations was tested using the *anova.cca* function with 9999 permutations.

To explore the association of genetic composition with local climate within the isolation by environment (IBE) model, we first selected potentially relevant climatic variables to avoid overfitting and collinearity in the subsequent dbRDAs. To do this, we applied the forward selection procedure using the *ordiR2step* function in the *vegan* R package based on the significance test with 9999 permutations and the adjusted *R*‐squared. The tested dataset included climatic variables that were identified as exercising a selective pressure on the beech distribution and genetic variation (Capblancq, Morin, et al., 2020; Pluess et al., 2016). These variables include temperature (the maximum “bio5” and the minimum “bio6” temperature), precipitation (annual precipitation “bio12”, precipitation of wettest month “bio13” and driest month “bio14”), evapotranspiration (annual potential evapotranspiration “annualPET”), and drought (aridity index “aridityIndex” and relative wetness to aridity “climaticMoistureIndex”) indicators. The bioclimatic variables were downloaded from the CHELSA, and evapotranspiration and drought indicators from the ENVIREM (Title & Bemmels, 2018) at a 30 arc‐sec resolution. All variables were standardized before variable selection using R *scale* function. At the end, four climatic variables (bio5, bio13, aridityIndexThornthwaite, and annualPET) were retained into the final dbRDA models.

To test for the effect of isolation by distance (IBD), we calculated a matrix of the Euclidean geographical distances among sampled populations (*geo*.) estimated from a raster layer depicting a “flat” landscape using QGIS. The current migration matrix was estimated using the divMigrate method (Sundqvist et al., 2016) in the *diveRsity* R package (Keenan et al., 2013).

The circuit theory within the isolation‐by‐resistance (IBR) model was applied to test the effect of topographic heterogeneity (*top*.) on genetic connectivity. Circuitscape 4.0 (McRae et al., 2016) was used to calculate pairwise landscape distances computed on resistance surface among all analyzed populations based on a terrain ruggedness index derived from the digital elevation model in QGIS.

Finally, we examined the past evolutionary history of Oriental beech as one of the causative factors contributing to the contemporary genetic composition in terms of isolation by colonization (IBC). We used the ancestry coefficient (*Q*‐value) obtained from the STRUCTURE analysis for *K* = 3 (see Results), assuming that the genetic ancestry (*anc*.) was an appropriate proxy of the postglacial colonization history of the species. We conducted a principal component analysis (PCA) on the set of *Q*‐value and retained the first two PCs obtained using the *prcomp* R function (R Core Team, 2022).

Before dbRDA analyses, the dissimilarity matrices (*top*., *geo*., *mig*.) were transformed into vectors using the principal coordinates of neighbor matrices (PCNM; Borcard & Legendre, 2002) with the *pcnm* function in the *vegan* R package. Only the first score components were retained in downstream analyses. The explanatory variables included in the final models were scaled and checked for multicollinearity using *scale* and *corr* R functions (R Core Team, 2022), respectively; the correlation matrix was visualized using *corrplot* R package (Wei & Simko, 2021; Appendix S3: Figure S3.2).

## 3 RESULTS

### 3.1 Diversity and differentiation

In total, 293 alleles were detected with an average of 22.54 alleles per locus (Appendix S3: Table S3.2). No evidence of linkage disequilibrium between each pair of loci across the population was detected.

The expected heterozygosity (*H*_E_) was very similar across all populations, reaching an average of 0.687 (Table 1). At the regional level, *H*_E_ was significantly lower in the Hyrcanian populations (0.654; *p* < .05) than in other regions. The populations from the Lesser Caucasus were characterized by having the highest gene diversity (*H*_E_ = 0.706). Allelic richness (*A*_R_) ranged from 5.366 (HZ_02) to 7.936 (LC_05) with a mean on 8.189 and was again significantly lower in the Hyrcanian populations (5.878; *p* < .001; Figure 2, Table 1). In most populations, private alleles were detected, with the highest average number noted in the Hyrcanian stands (3.000) and the lowest in populations from the Eastern Caucasus (AZ; 0.909).

**TABLE 1:** Summary of genetic diversity parameters of Oriental beech (AZ, Azerbaijan; GC, Greater Caucasus; HZ, Hyrcanian stands; LC, Lesser Caucasus) and results of the M‐ratio test under the two‐phase model (TPM) estimated for sampled populations.

ID	*A*	*A*_R_	*P*_A_	*H*_O_	*H*_E_	*F*_IS_	*F*_IS_Null	Null	MR	MReq	*p*‐Value
GC_01	8.692	7.312	3.000	0.620	0.705	0.130	0.025	0.066	**0.606**	**0.744**	**.0477**
GC_02	9.154	7.409	1.000	0.618	0.710	0.131	0.024	0.055	0.615	0.746	.0733
GC_03	8.000	7.280	1.000	0.618	0.694	0.122	0.038	0.075	**0.582**	**0.749**	**.0052**
GC_04	8.692	7.197	0.000	0.659	0.681	0.018	0.058**	0.027	**0.563**	**0.736**	**.0054**
GC_05	8.769	7.077	1.000	0.653	0.692	0.055	0.009	0.046	**0.553**	**0.745**	**.0201**
GC_06	8.077	7.000	1.000	0.581	0.681	0.135	0.032	0.069	**0.521**	**0.742**	**.0052**
GC_07	9.000	6.976	1.000	0.596	0.689	0.120	0.022	0.057	**0.527**	**0.746**	**.0001**
GC_08	9.923	7.557	2.000	0.682	0.708	0.025	0.024	0.027	0.628	0.735	.0732
GC_09	9.000	6.979	1.000	0.620	0.656	0.050	0.022	0.042	**0.538**	**0.748**	**.0067**
GC_10	9.077	7.553	2.000	0.647	0.722	0.116	0.027	0.061	**0.558**	**0.726**	**.0068**
GC_11	10.000	7.886	0.000	0.621	0.691	0.141	0.016	0.059	0.685	0.735	.3950
Greater Caucasus (average)	8.965	7.293	1.455	0.629	0.694	0.095	0.027	0.053	–	–	–
LC_01	7.154	7.003	3.000	0.690	0.701	0.011	0.012	0.024	0.596	0.713	.0745
LC_02	9.615	7.700	2.000	0.616	0.738	0.158	0.012	0.076	0.638	0.740	.1081
LC_03	9.769	7.893	2.000	0.651	0.709	0.080	0.018	0.047	0.683	0.744	.2075
LC_04	10.308	7.836	5.000	0.618	0.716	0.122	0.048	0.059	0.609	0.738	.0731
LC_05	10.077	7.936	2.000	0.662	0.714	0.052	0.023	0.040	0.619	0.729	.0636
LC_06	9.462	7.503	1.000	0.633	0.696	0.091	0.047	0.048	**0.558**	**0.739**	**.0031**
LC_07	9.923	7.294	1.000	0.613	0.689	0.079	0.035	0.054	**0.585**	**0.737**	**.0200**
LC_08	9.923	7.706	2.000	0.613	0.689	0.079	0.035	0.055	**0.585**	**0.737**	**.0201**
Lesser Caucasus (average)	9.529	7.609	2.250	0.637	0.706	0.084	0.029	0.050	–	–	–
HZ_01	8.385	6.391	4.000	0.546	0.649	0.205	0.091**	0.042	**0.597**	**0.751**	**.0052**
HZ_02	6.692	5.366	2.000	0.596	0.658	0.093	0.016	0.061	**0.590**	**0.767**	**.0012**
Hyrcania (average)	7.538	5.878	3.000	0.571	0.654	0.149	0.053	0.051	–	–	–
AZ_01	8.077	6.790	1.000	0.659	0.667	−0.003	0.025	0.021	**0.554**	**0.729**	**.0032**
AZ_02	8.308	7.225	1.000	0.608	0.680	0.111	0.061**	0.052	0.600	0.731	.0738
AZ_03	8.385	7.127	0.000	0.624	0.700	0.104	0.015	0.068	**0.549**	**0.734**	**.0130**
AZ_04	7.154	6.728	1.000	0.658	0.686	0.021	0.010	0.031	**0.530**	**0.730**	**.0023**
AZ_05	8.231	6.495	1.000	0.563	0.634	0.102	0.023	0.075	**0.549**	**0.747**	**.0041**
AZ_06	8.538	6.896	3.000	0.600	0.674	0.097	0.029	0.057	**0.541**	**0.737**	**.0135**
AZ_07	9.077	7.119	1.000	0.641	0.687	0.061	0.036**	0.037	0.609	0.741	.0638
AZ_08	8.154	7.084	1.000	0.621	0.686	0.085	0.032	0.052	**0.592**	**0.724**	**.0463**
AZ_09	8.538	7.026	0.000	0.616	0.685	0.105	0.012	0.063	**0.593**	**0.737**	**.0088**
AZ_10	8.385	6.557	0.000	0.591	0.652	0.085	0.021	0.054	**0.586**	**0.749**	**.0030**
AZ_11	7.923	6.466	1.000	0.576	0.653	0.120	0.037	0.075	**0.597**	**0.748**	**.0342**
Azerbaijan (average)	8.252	6.865	0.909	0.614	0.673	0.081	0.027	0.053	–	–	–
Average across all populations	8.743	8.189		0.622	0.686	0.123	0.020	0.059	–	–	–

*Note*: Significant values of M‐ratio and *p*‐value are in bold.

Abbreviations: *A*, the average number of alleles; *A*_R_, allelic richness based on minimum sample size; *F*_IS_, inbreeding coefficient; *F*_IS_Null, inbreeding coefficient with “null alleles” correction and Null – null allele frequency; *H*_E_, expected heterozygotes; *H*_O_, observed heterozygotes; MR, the mean observed M‐ratio; MReq, the M‐ratio generated under mutation‐drift equilibrium; *P*_A_, number of private alleles; *p*‐value, the probability of significant test for the deficiency in M‐ratio based on Wilcoxon signed‐ranks test; **, observed deficiency of heterozygotes may result from inbreeding; *, deviation from Hardy–Weinberg equilibrium at *p* < .05.

Significant homozygotes excess (*p* < .05) was detected in all populations except for LC_01 and AZ_07. The inbreeding coefficient (*F*_IS_) ranged from −0.003 to 0.205 with a mean on of 0.123 and did not differ significantly among regions (*p* = .368). *F*_IS_ estimated with a “null alleles” correction (*F*_IS_Null) was lower in most of the populations (0.020 on average). The presence of null alleles (ca. 6% on average) was indicated as the likely factor of homozygosity excess; in four populations inbreeding likely had a substantial impact on this estimation (Table 1).

The global *F*_ST_ estimated with the ENA correction reached a significant value of 0.033 (95% CI: 0.028–0.039). Pairwise *F*_ST_ ranged from −0.002 to 0.137 and was significantly higher than zero in all populations (Appendix S3: Table S3.3). The most divergent were Hyrcanian and two Azerbaijan (AZ_12 and AZ_13) populations. At the regional scale, the lower *F*_ST_ were observed within Greater (GC; *F*_ST_ = 0.006) and Lesser (LC; *F*_ST_ = 0.012) Caucasus populations, while the Hyrcanian (HZ) and Azerbaijan (AZ) stands were moderately differentiated (*F*_ST_ = 0.036 and *F*_ST_ = 0.034, respectively). A significant difference among the geographic regions in terms of pairwise *F*_ST_ was inferred (*p* < .05). The highest *F*_ST_ were found between the West‐Central Caucasus and Hyrcanian populations (*F*_ST_ = 0.097), and the latter with the Azerbaijan group (*F*_ST_ = 0.075), while the lowest between West‐Central Caucasus and Azerbaijan (*F*_ST_ = 0.011).

### 3.2 Population structure

**FIGURE 3:**
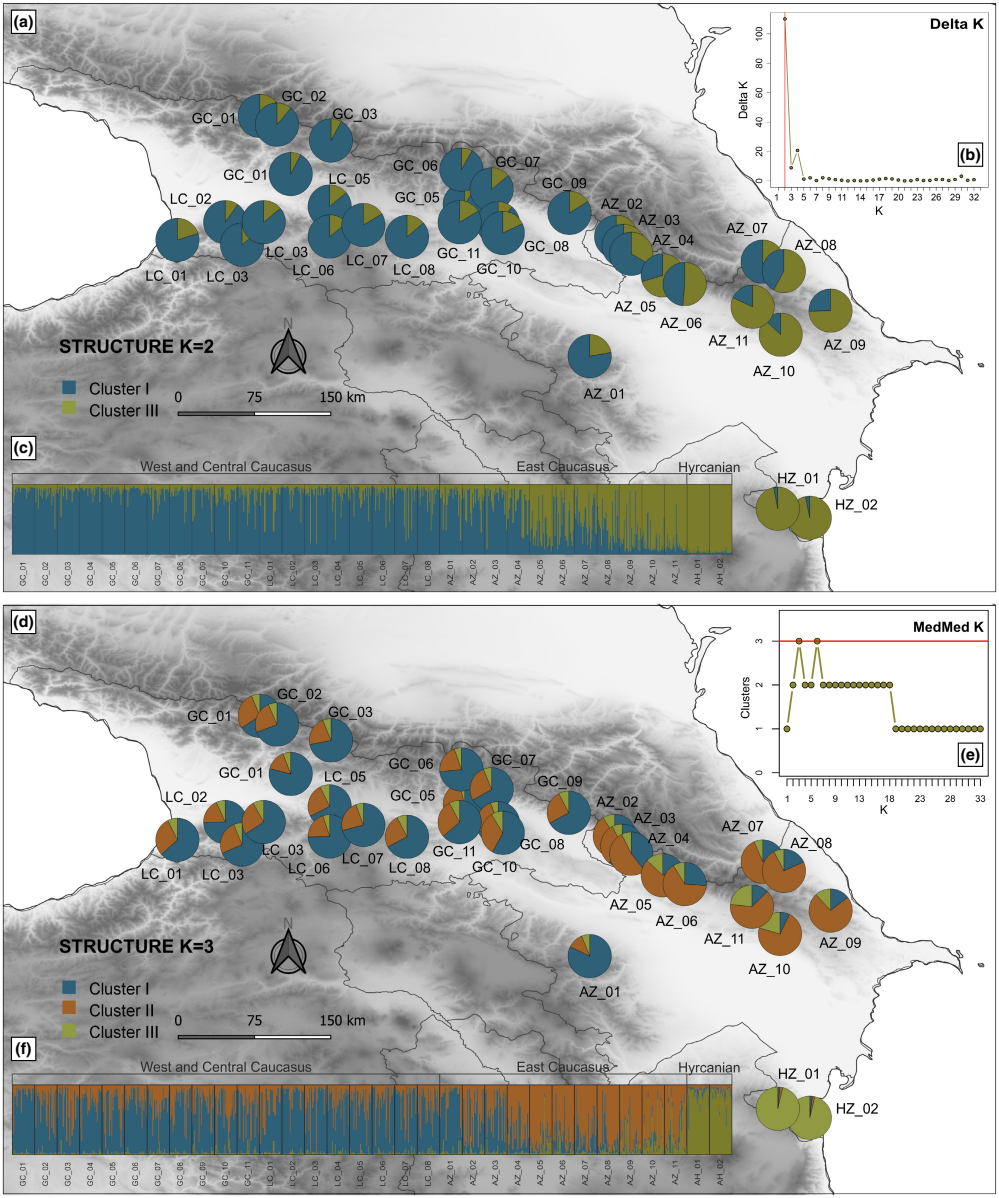
Spatial genetic structure estimated for the Oriental beech populations across the South Caucasus based on nSSRs using STRUCTURE for *K* = 2 (above) and *K* = 3 (below). Pie charts represent the genetic ancestry of each population across the study site (a and d). Admixture assignment of each individual to the inferred *K* clusters was visualized as barplots; each bar denotes the individual proportion of each of the detected genetic lineages (c and f). *K*‐selection plots according to Evanno's method (2005, b) and Puechmaille (2016, e) approaches show the highest value at *K* = 2 and *K* = 3 as the most likely number of clusters, respectively. Population abbreviations as in Table 1; Appendix S1: Table S1.1.

According to Δ*K*, the most supported number of genetic clusters was *K* = 2, which showed a west–east gradient of differentiation (Figure 3a–c). The most geographically widespread Cluster I contained all populations from the Greater (GC) and Lesser (LC) Caucasus (average *Q* = 87%), and also the populations located in the western part of Azerbaijan (AZ_01–04) with relatively high *Q*‐values reaching >75%. The remaining Azerbaijan populations (AZ_05–11) were mostly placed in Cluster II (average *Q* = 67%) together with the Hyrcanian stands (HZ_01–02, average *Q* = 97%) that showed the most limited genetic admixture to the remaining populations.

Although the second‐best group was *K* = 4 (Figure 3b), which was also supported by the Ln Pr (*X*|*K*) method (Appendix S3: Figure S3.3); this was not considered to be as informative. Two of the inferred clusters did not reach a mean threshold value of *Q* > 0.5 in all populations assigned to given groups, pointing to the presence of a spurious cluster. Consequently, we considered a clustering result of *K* = 3, which seems to be justified biologically and showed a clear geographical coherence (Figure 3d,e). This revealed a further substructure of the Azerbaijan populations, splitting populations into two groups, roughly consistent with the north–south pattern of differentiation. However, relatively high admixture across the study sites was observed, mostly among the West‐Central and Azerbaijan populations, except for the most distinct ones (Figure 3e,f).

All the Hyrcanian stands and also most of the populations from the Greater Caucasus (except for GC_02, CG_08 and CG_11) and Azerbaijan (except for AZ_2 and AZ_07) showed signs of a significant bottleneck (Table 1). However, only three populations from the Lesser Caucasus (LC_06‐CL_08) experienced demographic fluctuations.

### 3.3 Drivers of genetic differentiation

Among all tested GLMs (Table 2), the model incorporating the distance from the putative LGM refugium (Dist_LGM_) had the highest support. For both *A*_R_ and *H*_*E*_, the Dist_LGM_ model consistently had the highest Akaike weights (*w*_i_ >0.68) and Nagelkerke *R* ^2^’s (.665 and .499, respectively), pointing to west–east decreasing patterns (Figure 2).

**TABLE 2:** Summary statistic of the generalized linear models (GLMs) of genetic diversity metrics (*A*_R_, allelic richness and *H*_E_, expected heterozygosity) against the current habitat suitability (HS_CURR_), distance from putative LGM refugium (Dist_LGM_), genetic admixture (*G*_ADMIX_), latitude, and longitude.

Model	Nagelkerke *R* ^2^	Estimate	Pr(>|*t*|)	AIC	*w*_i_	ΔAIC
*A*_R_ ~ HS_CURR_	.348	1.978	<.01	46.39	0.001	13.53
***A***_**R**_ **~ Dist**_**LGM**_	**.665**	**−0.002**	**<.001**	**32.86**	**0.810**	**0**
*A*_R_ ~ *G*_ADMIX_	.002	−0.193	.823	56.63	0	23.76
*A*_R_ ~ latitude	.584	0.383	<.001	36.89	0.118	4.03
*A*_R_ ~ longitude	.584	−0.164	<.001	36.89	0.081	4.59
*H*_E_ ~ HS_CURR_	.277	0.086	<.01	−155.12	0.002	11.77
***H***_**E**_ **~ Dist**_**LGM**_	**.499**	**0.000**	**<.001**	**−166.9**	**0.681**	**0**
*H*_E_ ~ *G*_ADMIX_	.018	−0.027	.467	−145.3	0	21.55
*H*_E_ ~ latitude	.304	0.304	<.001	−156.33	0.003	10.55
*H*_E_ ~ longitude	.474	−0.007	<.001	−165.33	0.314	1.55

*Note*: The best models according to Akaike information criterion (AIC) and Akaike weights (*w*_i_) are in bold.

**FIGURE 4:**
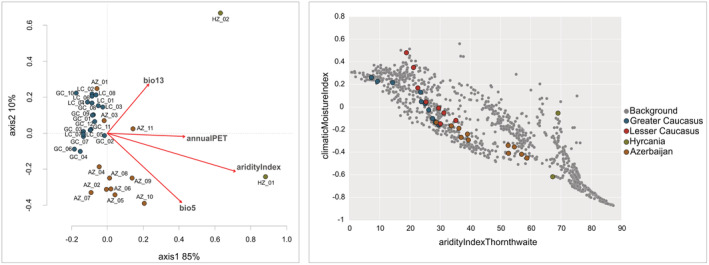
Projection of populations and environmental variables along the first two dbRDA axes (left panel) and diagram of the ecological requirements of Oriental beech in terms of aridity (aridityIndexThornthwaite) and relative wetness to aridity (climaticMoistureIndex; right panel). Studied sites (denoted with additional colors) were plotted against all remaining sites from the whole natural range (gray points). Population abbreviations as in Table 1; Appendix S1: Table S1.1.

According to partial dbRDA, migration, topographic heterogeneity, and geographic distance had an insignificant contribution to the structuring of the species' genetic variation (*p* > .05; Table 3). After excluding these factors, the full dbRDA model including climate, geographic distance, and ancestry produced a strong significant association (adj*R* ^2^ = .914; ≤.001), explaining 74% of the total variance (Table 3). The different partial dbRDA identified that 23% of this explained variance was associated with the pure effect of ancestry (17%; ≤0.001), and climatic variation (6%; ≤0.001). After excluding geographic distance, ancestry and climate significantly explained 73% of the total variation. (Table 3). Genetic ancestry (IBC) still explained the highest proportion of genetic variation, accounting for 17% even when controlling for confounding effects of other variables, while climate variation (IBE) explained 7% of the total variance (Table 3). The dbRDA plot indicated a significant association of genetic variation with the climatic variables among Hyrcanian (HZ_01 and HZ_02) and most of Azerbaijan (AZ) stands (Figure 4), showing their divergence from the remaining populations. We found that the two first axes explained most of the genetic variance among the populations (81% in total). dbRDA1 was mostly correlated with aridityIndex and annualPET, while dbRDA2 with bio5 and bio13 (Figure 4).

**TABLE 3:** Distance‐based redundancy analysis (dbRDA) to partition among‐population genetic variation (*F*_ST_) in Oriental beech and look into the effect of a set of explanatory variables, including climate (*clim*.), geography (*geo*.), genetic ancestry (*anc*.), topography heterogeneity (*top*.), and recent migration (*mig*).

Model	adj*R* ^2^	*p* (>*F*)	Proportion of explained variance	Proportion of unexplained variance	Proportion of confounded variance
**Full model:**	**.956**	**.762**	**0.** **238**		–
*F*_ST_ ~ *clim*. + *anc*. + *mig*. + *top*. + *geo*.					
Pure geography (IBD):	.01	.722	0.001	0.211	0.783
*F*_ST_ ~ *geo*. |(*clim*. + *anc*. + *mig*. + *top*.)					
**Pure ancestry (IBC):**	**.193**	.0001***	**0.118**	**0.211**	**0.671**
*F*_ST_ ~ *anc*. |(*clim*. + *mig*. + *top*. + *geo*.)					
Pure migration:	.032	.076	0.019	0.211	0.77
*F*_ST_ ~ *mig*.|(*clim*. + *anc*. + *top*. + *geo*.)					
**Pure climat (IBE):**	**.098**	.0319*	**0.064**	**0.211**	**0.725**
*F*_ST_ ~ *clim*.|(*anc*. + *mig*. + *top*. + *geo*.)					
Pure topography (IBR_TC_)	.012	.674	0.007	0.211	0.782
*F*_ST_ ~ *top*.|(*clim*. + *anc*. + *mig* + *geo*.)					
Total unexplained: 0.211					
Total explained: 0.209					
**Full model:** *F*_ST_ ~ *clim*. + *anc*. + *geo*.	**.914**	**.0001** ***	**0.737**	**0.266**	
Pure geography (IBD): *F*_ST_ ~ *geo*.|(*clim*. + *anc*.)	.008	.721	0.006	0.236	0.758
**Pure ancestry (IBC):** *F*_ST_ ~ *anc*.|(*clim*. + *geo*.)	**.253**	.0001***	**0.169**	**0.236**	**0.595**
**Pure climate (IBE):** *F*_ST_ ~ *clim*.|(*anc*. + *geo*.)	**.083**	.044*	**0.063**	**0.236**	**0.701**
Total unexplained: 0.236					
Total explained: 0.238					
**Full model:** *F*_ST_ ~ *clim*. + *anc*.	**.908**	.0001***	**0.727**	**0.273**	
**Pure ancestry (IBC):** *F*_ST_ ~ *anc*.|(*clim*.)	**.244**	.0001***	**0.170**	**0.242**	**0.588**
**Pure climate (IBE):** *F*_ST_ ~ *clim*.|(*anc*.)	**.09**	.0118*	**0.071**	**0.242**	**0.687**
Total unexplained: 0.242					
Total explained: 0.241					

*Note*: The significant models are in bold.

***p < .001; *<.05.

### 3.4 Ecological niche modeling

SDMs showed high levels of predictive performance with a similar score of AUC, reaching >0.946. The precipitation of the warmest quarter (bio18) and the temperature seasonality (bio4) were defined as the most important variables limiting the distributional patterns of the species with a relatively high contribution of >68% and >16%, respectively, in all tested models (Table 4).

**TABLE 4:** Predictive performance of the species distribution models (SDMs) evidenced by the area under the curve (AUC) values, relative contribution (%) of selected bioclimatic variables to models (bold indicates the higher scores), and potential geographical areas estimated using different threshold values of habitat suitability (%) and altitudinal range for Oriental beech at current (1960–1990), Last Glacial Maximum (LGM; ca. 21 ka BP) and future climatic scenarios (RCP; ca. 2071–2100), SDMs conducted across the range*.*

SDMs	Current	LGM	RCP4.5	RCP8.5
Area under the curve (AUC)	0.946	0.948	0.949	0.946
Variable contribution				
Annual mean temperature (bio 1)	1.6	0.8	1.8	1.5
Isothermality (bio 3)	0.5	0.3	0.6	0.5
Temperature seasonality (bio 4)	**16.2**	**16.1**	**16.2**	**16.8**
Mean temperature of wettest quarter (bio 8)	7.3	6.4	5.5	5.2
Mean temperature of driest quarter (bio 9)	0.2	0.9	0.5	0.5
Precipitation seasonality (bio 15)	0.4	0.5	0.3	0.4
Precipitation of warmest quarter (bio 18)	**68.9**	**70.2**	**68.4**	**70.0**
Precipitation of coldest quarter (bio 19)	5.0	4.8	6.6	5.1
Average altitude (m a.s.l.)	710	534	930	1485
Suitability area (10^3^ km^2^)				
Low (15–29%)	245	141	148	146
Medium (30–59%)	401	245	274	157
High (60–74%)	212	49	129	58
Very high (75–100%)	79	55	150	32
Total area	937	490	701	392

**FIGURE 5:**
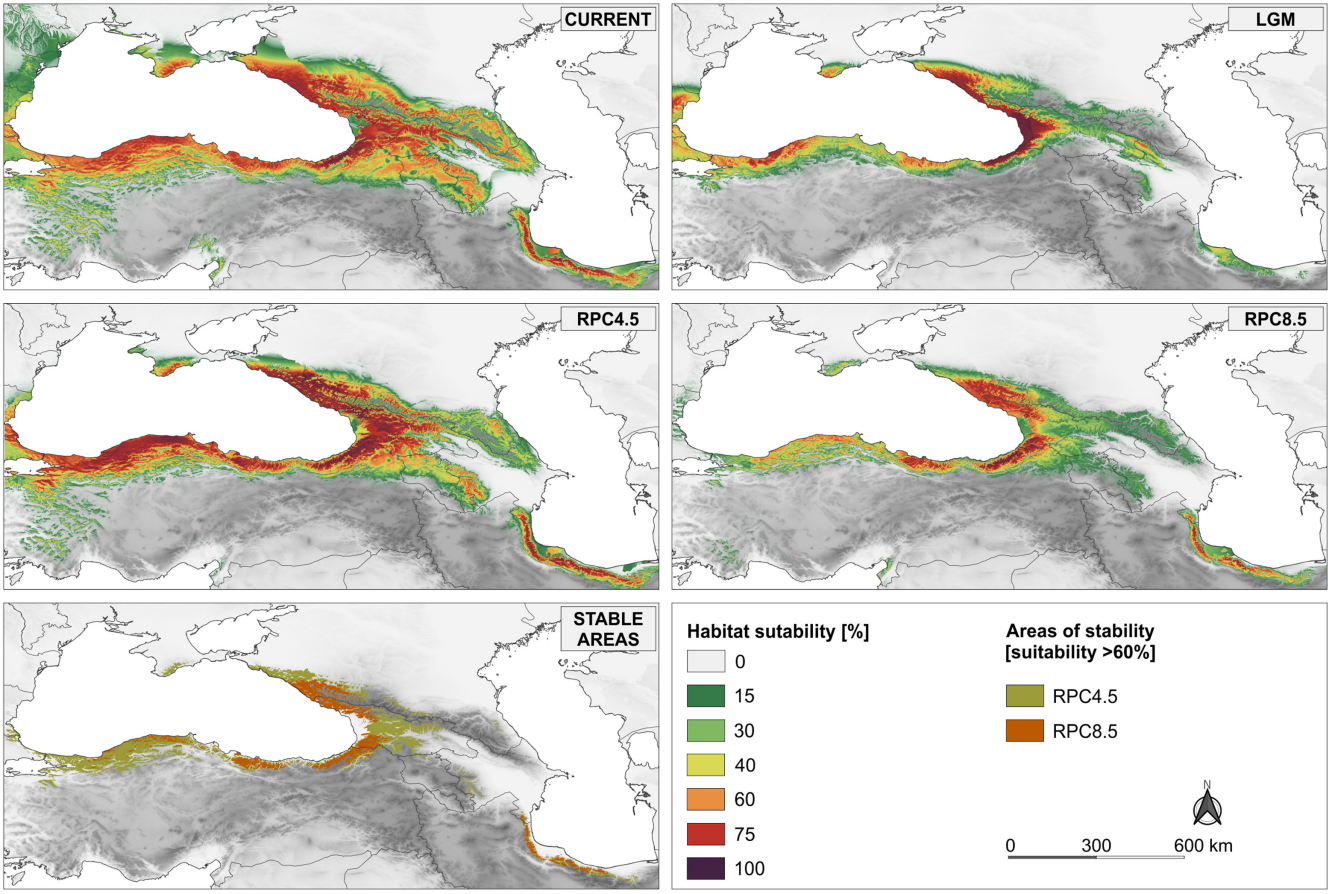
Species distribution modeling for Oriental beech based on climatic variables, projected at current (ca. 1981–2010), the Last Glacial Maximum (LGM; ca. 21 ka BP), and future (RCP4.5 and RCP8.5; ca. 2071–2100) climatic scenarios. Climatically suitable areas for the species are defined using the maximum entropy algorithm implemented in Maxent. The areas of stability for the species defined as a region of overlap between the projected future (RCP4.5 and RCP8.5).

The distribution model under the current climatic conditions properly described the present range of Oriental beech (Figure 5). During LGM, most of the Caucasus region was climatically unsuitable for the species, and potentially suitable conditions existed in three main areas: the most eastern part of the Pontic Mts. (Turkey) with the Adjara region (suitability >75%), the Colchis area with Abkhazia, and the adjacent part of Russia (suitability >75%), and to some extent the Hyrcanian region (suitability 40%). Apart from those refugial areas, the Iori Plateau in southeastern Georgia and the north‐western part of the Greater Caucasus in Azerbaijan seemed to offer suitable habitats for the species with a relatively high suitability score reaching 40–75%. Furthermore, the residual areas in the Ganja‐Gazakh and Karabakh regions in Azerbaijan were also indicated as climatically suitable for the species but with lower support (<40%). Beyond the Caucasus, the favorable area of occurrence with the higher support (>65%) was also predicted to cover the remaining part of the Pontic Mts. (Turkey) and Crimea.

**FIGURE 6:**
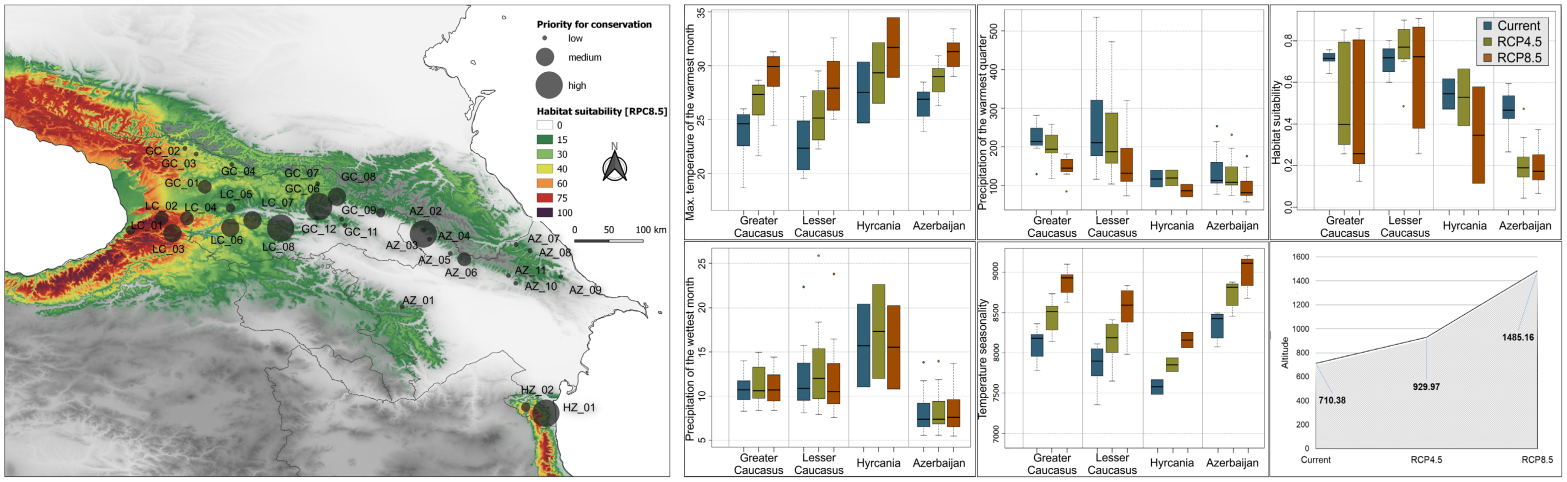
Populations of Oriental beech with priority for conservation inferred with reserve selection algorithm implemented in DIVA‐GIS in relation to the potential future distribution under the pessimistic scenario (RCP8.5, ca. 2070). Disk diameters are proportional to the value of genetic parameters, following the figure legends (left panel). Bar charts presenting bioclimatic variables with the highest contribution in SDMs, variables significant association with genetic structure, habitat suitability and average shifts in elevation for the current and future projections (right panel). Population abbreviations as in Table 1; Appendix S1: Table S1.1.

Regarding the future predictions under RCP4.5, significant changes in habitat suitability in the Hyrcania and western part of the species range are not expected (Figure 5). Nevertheless, a reduction in area of climatically stable areas was predicted in Azerbaijan, particularly in the western part of the Greater Caucasus, where some areas may be completely unsuitable. However, more drastic contraction of the species range was predicted under RCP8.5. Almost 69% of the current areas with suitability >60% might be lost (Figure 5), and only some areas in the Pontic Mts., Adjara region and Abkhazia with the adjacent part of Russia remain as climatic refugia for the species in the future. This would mainly affect the central‐eastern parts of the species range in the South and North Caucasus, and the western part of Pontic Mts. Moreover, upward shifts of the species range are predicted, reaching a mean of 930 m and 1485 m a.s.l., respectively (Figure 6; Table 4). Stable climatic refugia for Oriental beech in future remain only in the Colchis area with Abkhazia, the adjacent part of Russia, the Hyrcanian region and the East Pontic Mts.

## 4 DISCUSSION

### 4.1 Spatial genetic pattern: Implications for the postglacial history

Our genetic data located the main LGM refugia for Oriental beech in areas already identified in the Caucasus (Connor & Kvavadze, 2009; Dagtekin et al., 2020; Tarkhnishvili et al., 2012). Additionally, the data showed that the intraspecific divergence in the species was mostly a result of the climate‐driven vicariance process. Specifically, the most widespread group, in the west‐central South Caucasus, represents genetic lineages derived from refugial areas in the Colchis, while the most spatially restricted clusters concentrated in the eastern South Caucasus correspond to the Hyrcanian refugium. The detected Caucasian‐Hyrcanian genetic split in Oriental beech is similar to that reported previously for other plants and animal species (Christe et al., 2014; Ibrahimov et al., 2010; Maharramova et al., 2018; Tarkhnishvili, 2014).

Given the detected significant eastwards gradient of genetic diversity, we can conclude that the Colchis refugium likely acted as the major source of postglacial expansion. Notably, the west–east genetic pattern seems to be also consistent with the assumptions of the leading‐edge model of range expansions related to density‐dependent processes (Hampe & Petit, 2005). This pattern has been previously documented for trees that have undergone a long‐distance migration pattern (Roberts & Hamann, 2015). Given the general assumptions regarding the refugial area (Hewitt, 2000), the high allelic diversity and private alleles found in populations from the Adjara‐Imereti Range (southwestern Colchis) support the location of refugia there. Similarly, populations from the Talysh Mts. contain a unique genetic variation, suggesting the putative long‐term and isolated presence of the species in that area. The palynological records support the persistence of Oriental beech at lower elevation in the Alborz Mts. during the LGM (Ramezani & Joosten, 2015; E. Ramezani et al., personal communication) but not in the Talysh Mts., which was also suggested by the SDMs results (Figure 5; Dagtekin et al., 2020).

Climatically suitable areas during the glacial phase were additionally revealed in the Iori Plateau, Alazani valley, and some gorges of the western Azerbaijan part of the Greater Caucasus (suitability >60%). Palaeobotanical evidence that Oriental beech survived in the eastern part of the South Caucasus during the LGM is lacking (Connor, 2006) but early‐Holocene population remnants found in the Iori region (eastern Georgia; Gogichaishvili, 1984) suggest that local persistence in this area is plausible. Genetic distinction of populations from the Azerbaijan part of the Greater Caucasus with mixed ancestry at *K* = 3, and high allelic richness in some populations from the Gombori Range (GC_08–11) and Shaki Region (AZ_06) strengthened the hypothesis on the refugial areas there. Additionally, SDM indicated the local foci of the species occurrence in the Ganja‐Gazakh and Karabakh regions (<30% of suitability) in Azerbaijan, which needs further genetic confirmation.

The high genetic link of the populations from the Daghlig Shirvan region (AZ_10 and 11) to the Hyrcanian gene pool suggests the possible contribution of the Hyrcanian refugium to the colonization of Oriental beech in the eastern South Caucasus. However, the extremely arid conditions that have prevailed in the Kura‐Araz region (eastern Caucasus) since the LGM (Leroy et al., 2013) might have acted as an ecological barrier hindering the postglacial colonization of the eastern Caucasus by seeds from the Hyrcanian source. Moreover, the dual colonization of the eastern part of the Caucasus should imply the formation of a secondary contact zone (Rius & Darling, 2014), which is not observed in the eastern Caucasus. Hence, given the spatial scale of the studied area, we postulate that contribution of the Hyrcanian source resulted from efficient pollen‐mediated gene flow between both refugia. Indeed, long‐distance pollen dispersal, distances even up to 1000 km, is frequent in beech (Belmonte et al., 2008; Piotti et al., 2012). A similar pattern of the efficient pollen‐mediated gene flow among distinct refugia during postglacial expansion has been shown for *Abies alba* (Liepelt et al., 2002; Piotti et al., 2017) and *Pinus banksiana* (Godbout et al., 2010). Wider sampling in the East Caucasus would shed light on the postglacial migration in this area.

### 4.2 Drivers of genetic and differentiation patterns

Considering the postglacial history of the Oriental beech mostly related to single Colchis refugium, and environmental gradients present in the study area, it seems that our inference is burdened by uncertainty due to the correlation of spatial‐environmental factors. However, we were able to disentangle the forces structuring neutral genetic diversity across species' ranges, applying the variance partitioning approach that reduces the confounding effects of potential spatially correlated predictors and quantifies its relative influence (Legendre & Legendre, 2012).

Considering the topographic complexity of the Caucasus and the IBC hypothesis that involves both historical and adaptation processes as drivers of the population's divergence (Orsini et al., 2013), we expected to find strong geographically structured diversity with a clear split between the Greater and Lesser Caucasus. However, we found no support for topographic complexity being an important factor in genetic structure. Genetic distinctiveness between these mountain ranges for another wind‐pollinated tree, *Pinus sylvestris* (Dering et al., 2021), has been explained by the direction of prevailing winds in the region and potential local adaptation. The homogenization of the beech's gene pool across the region is likely due to effective pollen‐mediated gene flow. Indeed, we observed very low overall genetic differentiation for Greater Caucasus (*F*_ST_ = 0.006) and Lesser Caucasus (*F*_ST_ = 0.012) populations.

The clear west–east gradient of genetic differentiation in Oriental beech could suggest a strong pattern of IBD. However, given that IBD does not account for the landscape heterogeneity (Jenkins et al., 2010) and can interfere with the alternative patterns of population structure resulting from colonization history and landscape resistance to gene flow (Orsini et al., 2013; van Strien et al., 2015), this seems to be an unrealistic scenario due to the oversimplification of processes involved. Accordingly, after controlling for the confounding effect of genetic ancestry and climate, the geographic distance by itself had comparatively little contribution to the observed genetic pattern (1% of the total variation, *p* > .05). It seems that the mid‐elevation areas in the west‐central Caucasus and northern Azerbaijan part of the species range act as corridors for the extensive gene flow in the species. On the contrary, the finding that the Kura‐Araz lowland acts as a substantial barrier to gene flow among the Hyrcanian and the remaining Caucasian populations supports our assumption that the detected differentiation is structured by the environmental resistance and evolutionary history. Indeed, our results indicated that climate and ancestry explained the largest amount of among‐population variation (72%, *p* < .001) after omitting the insignificant effect of topography, geography, and migration. A significant proportion of the variation could be attributed exclusively to genetic ancestry that refers to the IBC model (17%).

In the absence of palaeobotanical evidence for a cryptic refugium in the eastern part of the South Caucasus that could act as a source of eastward colonization, we cannot conclusively state whether the distinct genetic composition of Azerbaijan populations is consistent with the IBC or IBE. Since postglacial colonization is able to generate patterns similar to IBE (Hampe & Petit, 2005; Orsini et al., 2013), it seems that detected divergence is a result of recent postglacial history rather than vicariance process in an isolated cryptic refugium. Additionally, we did not find the accumulation of private alleles and high genetic variation in the Azerbaijan populations, which could support the presence of a cryptic refugium in this area. Conversely, the initial pattern produced by vicariance might have been partly swamped by the current relatively high gene flow among west‐central Caucasus and Azerbaijan populations resulting in low genetic differentiation (*F*_ST_ = 0.011). Consequently, the current distinctiveness of the Azerbaijan sites could be a weak signal of the initial founder effect originating from the colonization stage. According to IBE model, the selection against maladapted migrants may allow the genetic signal of the initial structure to be preserved in the neutral diversity for generations (Orsini et al., 2013).

The divergence of the Hyrcanian and most of the Azerbaijan stands (AZ_04–AZ_11) could also have been caused by local adaptation, given the climatic distinctiveness of the East Caucasus. The detected significant signal of IBE, accounting for 6% of the total variation, suggests that the genetic composition is partially structured by local climate. Specifically, aridity, maximum temperature, precipitation of the wettest month, and annual potential evapotranspiration were significantly associated with genetic distance. According to the autecology diagram, these range‐edge populations can be considered as ecologically marginal (Figure 6). Such a distributional pattern implies the development of local adaptations. However, due to methodological constraints, our results are not a pertinent proxy of adaptive divergence, which requires the detection of genomic signals of adaptation. Nevertheless, the selectively neutral markers may show some association with the environment due to genome hitchhiking leading to the IBE patterns (Nosil et al., 2008), which means that the hypothesis on the contribution of local adaptation to the genetic structure of Oriental beech remains valid. Indeed, a strong association between neutral genetic composition and environmental gradients has been found in other tree species (Muniz et al., 2022; Sork et al., 2010).

### 4.3 Conservation implication

In contrast to some studies (Dagtekin et al., 2020; Khalatbari Limaki et al., 2021), our SDM models are not so pessimistic about the future theoretical distribution of Oriental beech, especially in Turkish and Hyrcanian parts of the range. Nevertheless, much of the currently highly suitable areas for the species may be lost. The most prominent changes are the distributional contractions projected in the Azerbaijan part of the Greater Caucasus, Armenia, and eastern Georgia (Figure 5). Moreover, the range shifts westward and may show a twofold increase in elevation under the most pessimistic scenario (Figure 6). The shifting to the higher elevation of the species can be mostly explained by temperature increases because other climatic trends (e.g., precipitation) are not generally related to elevation. According to the climate projection, the mean temperature in the Caucasus Mts. is expected to rise by at least 3°C by the end of this century compared with the current condition. Higher temperatures are assumed to increase the intensity of soil drought due to the forcing effect on potential evapotranspiration (Bergh et al., 2003). Further decreased precipitation by 33% (bio18) may exacerbate soil water deficit impacting the species' growth at lower elevations forcing it to track favorable conditions at higher elevations. The climate‐induced potential elevation shift of Oriental beech has also been reported for the Hyrcanian part of the species range (Khalatbari Limaki et al., 2021). The discrepancies among our results and previously presented SDMs (Dagtekin et al., 2020; Khalatbari Limaki et al., 2021) are likely due to the improved methodology used here. Climate rasters that fail to capture the effects of topography on microclimate may affect the accuracy of the predictions (Gavin et al., 2014; Karger et al., 2017). To reduce this uncertainty, we used climatic data from CHELSA that has higher accuracy in mountain‐specific conditions (Brown et al., 2018; Karger et al., 2017). Additionally, using the occurrence dataset drawn from map grid cells, as was done in Dagtekin et al. (2020), can be a source of model bias (Konowalik & Nosol, 2021).

Generally, the Caucasian populations of Oriental beech harbor relatively high neutral genetic variability, similarly to stands in Iran (Salehi Shanjani, 2011). However, in the context of habitat loss, the genetic resources of the species may be greatly impoverished, ultimately affecting its adaptive potential and thus the stability and resilience of forests in the region. Under climate change, efforts to conserve and manage species/biodiversity should focus on identifying climate change refugia (Barrows et al., 2020; Fady et al., 2016; Hoban et al., 2021; Keppel et al., 2015). Here, by integrating the landscape genetic analysis and ecological niche modeling, we were able to indicate the potential areas where the species may persist under projected climate change. Our results concurrently point out that areas located in the Colchis region, considered as long‐term climatic refugia for Oriental beech during the LGM, may also be efficient in supporting the species in future. Consequently, those populations should be under conservation efforts to preserve them in situ, for example, by establishing protected areas or by including them into a network of gene conservation units (GCUs), similarly to the approach already applied to forest tree species by EUFORGEN in Europe. The guidelines for the minimum qualification criteria that must be met for GCU certification are available (Koskela et al., 2013). They can be directly applied also in the Caucasian populations of Oriental beech. Our results provide additional information regarding genetic diversity that can support the process of GCUs establishment. Moreover, in light of the assumption that species adaptation to climate change mostly relies on the standing genetic variation (Savolainen et al., 2013), special attention should be paid to the population from the Adjara‐Imereti Range. These populations host the highest and most unique neutral genetic diversity that is of crucial conservation and management priority and can be highly relevant for the future resilience of the species. Additionally, populations from the Trialeti Range (LC_06‐LC_08), Khevsureti (GC_06), Gombori Range (GC_08), and Azerbaijan stands (AZ_01 and AZ_05) should be preserved in the context of maintaining a high spectrum of genetic diversity needed for sustainable beech forest management. However, given the adaptive potential of range‐edge populations to climate change (Fady et al., 2016; Hampe & Petit, 2005; Rehm et al., 2015) and that the species ability to persist under such changes will be determined by the responses of the local populations (Aitken et al., 2008), the Oriental beech populations at the range‐edge should also be considered. Our SDM showed that the eastern Caucasian gene pool of the species is expected to be seriously vulnerable because of increases in temperature and aridity (Figure 6), especially the peripheral Azerbaijan populations that already occur in marginal conditions and display low gene diversity. The detected excess of inbreeding and signs of bottlenecks may suggest that adverse demo‐genetic processes are already present in these populations. On the other hand, these populations may potentially harbor important adaptive properties generated under such environmental constraints (Aitken et al., 2008; Rehm et al., 2015). Given the projected extreme decreases in precipitation in the eastern domain of the species range, the probable intensification of the stochastic genetic processes may pose a risk to that unique gene pool. Another possible consequence of the climate‐driven range shifts might be the loss of landscape connectivity, triggering strong genetic drift. Furthermore, the detected strongly asymmetric gene flow among the Georgian and Azerbaijan population may also have serious evolutionary consequences related to adaptation lags of range‐edge populations due to receiving maladaptive alleles (Aitken et al., 2008; Fréjaville et al., 2020).

## 5 CONCLUSIONS AND FUTURE DIRECTIONS

We are aware that a complete understanding of how ecologically marginal populations of Oriental beech may cope with climate change adaptation requires a detailed investigation including a genome‐environmental association approach to identify a signature of local adaptation and the recognition effect of gene flow on adaptation (Capblancq, Fitzpatrick, et al., 2020). The SDM assumes a genetic homogeneity, and incorporating the adaptive genetic variation in climate change vulnerability assessment could deliver more reliable projections. Our study is the first approximation of the potential risks involved in climate change and induces far‐reaching thinking about the need of applying management solutions dedicated to maintaining the genetic resources of Oriental beech (Fady et al., 2016).

This study enriches our understanding of the evolutionary history of Oriental beech and the forces that shape its neutral genetic composition in the South Caucasus. Nevertheless, several other questions remain unanswered, waiting for comprehensive sampling across the whole species range and implementation of more relevant landscape genomic and demographic approaches. We would like to know what is the adaptive genetic potential of Oriental beech, how is it distributed across the species' range, and how this can be helpful for the species in tracking future climate change. These issues are important because of the potential range reduction of one of the most valuable Caucasian tree species, with implications for forest management in Europe (Brang et al., 2016).

## AUTHOR CONTRIBUTIONS

**Katarzyna Sękiewicz:** Conceptualization (equal); data curation (lead); formal analysis (equal); investigation (lead); methodology (lead); project administration (supporting); supervision (lead); visualization (lead); writing – original draft (lead). **Irina Danelia:** Resources (equal); writing – review and editing (supporting). **Vahid Farzaliyev:** Resources (equal); writing – review and editing (supporting). **Hamid Gholizadeh:** Resources (supporting); writing – review and editing (supporting). **Grzegorz Iszkuło:** Investigation (supporting); resources (supporting); writing – review and editing (supporting). **Alireza Naqinezhad:** Resources (supporting); writing – review and editing (supporting). **Elias Ramezani:** Writing – review and editing (supporting). **Peter A. Thomas:** Resources (equal); writing – review and editing (supporting). **Dominik Tomaszewski:** Writing – review and editing (supporting). **Łukasz Walas:** Formal analysis (equal); writing – review and editing (supporting). **Monika Dering:** Conceptualization (equal); data curation (equal); funding acquisition (lead); project administration (lead); resources (equal); supervision (supporting); writing – review and editing (supporting).

## Supporting information


Appendix S1–S3

## Data Availability

The data that support the findings of this study, including genotypes, raw dataset used for landscape genetics analyses and SDMs output files are openly available in FigShare at [https://doi.org/10.6084/m9.figshare.20227263.v1]. The sources of the species occurrence used for SDMs analyses and the pairwise *F*_ST_ used in landscape genetics analyses are provided in Supporting Information.
