# Supplementary material for: Past climatic refugia and landscape resistance explain spatial genetic structure in Oriental beech in the South Caucasus
Source: Ecol Evol. 2022 Sep 20;12(9):e9320. doi: 10.1002/ece3.9320 (PMC9490144; doi:10.1002/ece3.9320)
Supplement: Supplementary file 1 — Appendix S1–S3 [file ECE3-12-e9320-s001.docx]

# Appendix S1: Sampling material and genetic data acquisition

**Table S1.1** Description of sampled natural populations of Oriental beech across the South Caucasus (GC – Greater Caucasus, LC – Lesser Caucasus, HZ – Hyrcanian stands; AZ – Azerbaijan)

| **ID** | **Locality** | **N** | **Latitude (N)** | **Longitude (E)** | **Elevation [m** **a.s.l.]** |
| --- | --- | --- | --- | --- | --- |
| **Greater Caucasus:** | | | | | |
| GC_01 | Georgia, Racha Range | 26 | 42.4019 | 43.0695 | 1145 |
| GC_02 | Georgia, Svaneti Range | 28 | 43.0352 | 42.7329 | 1648 |
| GC_03 | Georgia, Svaneti Range | 20 | 42.9405 | 42.9196 | 1940 |
| GC_04 | Georgia, Svaneti Range | 26 | 42.7679 | 43.5089 | 1458 |
| GC_05 | Georgia, Gombori Range | 29 | 42.0728 | 44.9661 | 1188 |
| GC_06 | Georgia, Khevsureti | 25 | 42.4507 | 44.9305 | 1213 |
| GC_07 | Georgia, Khevsureti | 30 | 42.2343 | 45.2598 | 954 |
| GC_08 | Georgia, Gombori Range | 30 | 41.8641 | 45.3299 | 1327 |
| GC_09 | Georgia, Alazani Range | 30 | 41.9708 | 45.9771 | 890 |
| GC_10 | Georgia, Gombori Range | 25 | 41.7598 | 45.3814 | 1105 |
| GC_11 | Georgia, Gombori Range | 29 | 41.8768 | 44.9110 | 1050 |
| **Lesser Caucasus:** | | | | | |
| LC_01 | Georgia, Achara-Imereti Range | 15 | 41.6843 | 41.8345 | 200 |
| LC_02 | Georgia, Achara-Imereti Range | 29 | 41.8786 | 42.3556 | 1873 |
| LC_03 | Georgia, Achara-Imereti Range | 29 | 41.6326 | 42.5387 | 1898 |
| LC_04 | Georgia, Achara-Imereti Range | 31 | 41.8785 | 42.7752 | 1570 |
| LC_05 | Georgia, Likhi Range | 28 | 42.0487 | 43.4942 | 999 |
| LC_06 | Georgia, Trialeti Range | 30 | 41.7212 | 43.4951 | 1861 |
| LC_07 | Georgia, Trialeti Range | 27 | 41.8468 | 43.8619 | 1370 |
| LC_08 | Georgia, Trialeti Range | 30 | 41.7178 | 44.3372 | 1281 |
| **Hyrcanian forest:** | | | | | |
| HZ_01 | Azerbaijan, Hyrcania | 30 | 38.6564 | 48.7278 | 228 |
| HZ_02 | Azerbaijan, Hyrcania | 30 | 38.7547 | 48.3789 | 1428 |
| **Azerbaijan:** | | | | | |
| AZ_01 | Azerbaijan, LC, Ganja | 23 | 40.4175 | 46.3288 | 1599 |
| AZ_02 | Azerbaijan, Greater Caucasus | 23 | 41.6796 | 46.6864 | 954 |
| AZ_03 | Azerbaijan, Greater Caucasus | 25 | 41.6194 | 46.6989 | 670 |
| AZ_04 | Azerbaijan, Greater Caucasus | 18 | 41.5327 | 46.7815 | 611 |
| AZ_05 | Azerbaijan, Greater Caucasus | 28 | 41.2976 | 47.1249 | 854 |
| AZ_06 | Azerbaijan, Greater Caucasus | 26 | 41.2026 | 47.3638 | 1246 |
| AZ_07 | Azerbaijan, Greater Caucasus | 30 | 41.4479 | 48.2147 | 990 |
| AZ_08 | Azerbaijan, Greater Caucasus | 24 | 41.3461 | 48.4454 | 710 |
| AZ_09 | Azerbaijan, Greater Caucasus | 27 | 40.9138 | 48.9544 | 1036 |
| AZ_10 | Azerbaijan, Greater Caucasus | 28 | 40.8053 | 48.2103 | 903 |
| AZ_11 | Azerbaijan, Greater Caucasus | 28 | 40.9326 | 48.0892 | 865 |

**Details on Microsatellite amplification:** Thirteen nuclear microsatellite markers (nSSRs) originally developed for *Fagus sylvatica* (Pastorelli et al. 2003; Pluess and Määttänen 2013) (Appendix 3, Table S1) were amplified in multiplex PCR reactions in a total volume of 10 μl with ca. 60 ng template DNA, 10×Silver Hot Buffer, 2.5mM MgCl2, 0.2mM dNTPs, and 0.5U Sliver Hot Polymerase (Syngen, Poland). The nSSRs used were combined in the following multiplexes, i.e.: Multiplex I: FS1-15, FS1-03, FS3-04, FS4-46 and FCM5; Multiplex II: fagsyl_001018, fagsyl_001217, fagsyl_003994, and fagsyl_007038; Multiplex III: fagsyl_003093, fagsyl_003849, fagsyl_000909, and fagsyl_006075. The thermal PCR conditions for Multiplex I were as follows: an initial denaturation at 95°C for 15 min; 28 cycles of denaturation at 95°C for 30 s, annealing at 60°C for 60 s, elongation at 72°C for the 60s and final elongation at 72°C for 8 min. A touchdown PCR protocol was applied for the remaining multiplexes, with initial denaturation at 94°C for 15 min, followed by seven touchdown cycles of denaturation at 94 °C for 45 s, annealing at 60°C for 60 s (−1°C/cycle) and extension at 72°C for 60 s; 28 cycles of denaturation at 94°C for 45 s, annealing at 53°C for 60 s and extension at 72°C for 60 s and a final extension at 72°C for 10 min.

Separation of the amplified fragments was done on an Applied Biosystems 3130 Genetic Analyzer (Thermo Fisher Scientific, Waltham, USA) with an internal size standard, GeneScan™ 600 LIZ®. GeneMapper 4.0 (Thermo Fisher Scientific, Waltham, USA) was used to score genotypes. To classify observed nSSRs allele sizes into representative discrete alleles the Raw2Gen (IJ Chybicki, Kazimierz Wielki University, Poland, unpubl. data) was used.

# Appendix S2: Occurrence data and species distribution modelling procedure

**Species occurrence data acquisition:** Occurrences data of Oriental beech were collected from the literature (see list of reference below), Global Biodiversity Information Facility (Occurrence Download https://doi.org/10.15468/dl.ymvqwt accessed via GBIF.org on 3 July 2020), EufGis (http://www.eufgis.org/), SouthEast Regional Network of Expertise and Collections (SERNEC; http://sernecportal.org/portal/index.php), National Herbarium of Georgia Institute of Botany, Ilia State University, and the Soviet Military Topographic Maps (at a scale of 1:50,000, Headquarters of Geodesy and Cartography under the Council of Ministers of the USSR. 1955-1976) and Hyrcanian Forest Vegetation Database (Gholizadeh et al., 2019). Initially, the datasets hosted 1,225 georeferenced records, however, to reduce spatial autocorrelation, only one occurrence point per grid cell (1×1 km) was considered, leading to 810 unique records (Fig. S1) used in final procedures. Data handling was done using QUANTUM GIS 3.24.0 'Tisler' (QGIS.org, 2022).


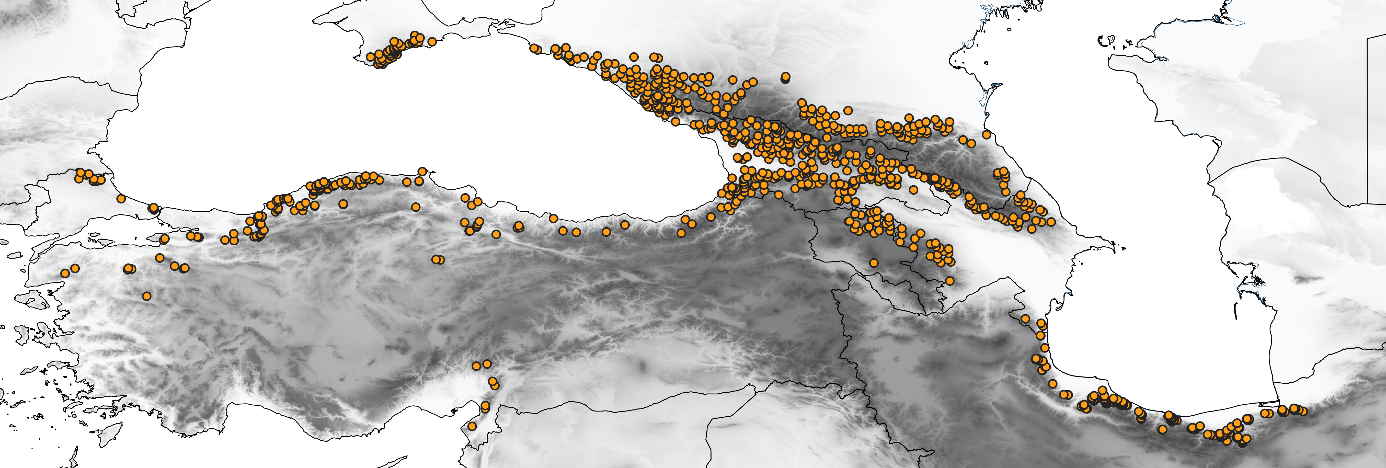
Figure S2.1 Occurrences data of Oriental beech used in the species distributions modelling

**Reference list of the species occurrence data:**

Akata, I., & Kumbasli, M. (2014). A new and rare record for Turkish *Cantharellus*. *7*, 143–145.

Akkemik, Ü., Caner, H., Conyers, G. A., Dillon, M. J., Karlioğlu, N., Rauh, N. K., & Theller, L. O. (2012). The archaeology of deforestation in south coastal Turkey. *International Journal of Sustainable Development & World Ecology*, *19*(5), 395–405. https://doi.org/10.1080/13504509.2012.684363

Alataş, M., Ezer, T., Kara, R., & Uyar, G. (2012). Abant Dağları’nda *Fagus orientalis* Lipsky. (Doğu Kayini) Ağaçlarının epifitik bryofitleri. *Bartın Orman Fakültesi Dergisi*, Special Issue, 98-105. https://dergipark.org.tr/tr/pub/barofd/issue/3393/46748

Amolikondori, A., Abrari Vajari, K., Feizian, M., & Di Iorio, A. (2020). Influences of forest gaps on soil physico-chemical and biological properties in an oriental beech (*Fagus orientalis* L.) stand of Hyrcanian forest, north of Iran. *IForest - Biogeosciences and Forestry*, *13*(2), 124. https://doi.org/10.3832/ifor3205-013

Arslan, B., Öztürk, Ş., & Oran, S. (2011). Lecanora, Phaeophyscia and *Rinodina* species new to Turkey. *Mycotaxon*, *116*(1), 49–52. https://doi.org/10.5248/116.49

Bayramzadeh, V., Attarod, P., Ahmadi, M. T., Ghadiri, M., Akbari, R., Safarkar, T., & Shirvany, A. (2011). Variation of leaf morphological traits in natural populations of *Fagus orientalis* Lipsky in the Caspian forests of Northern Iran. *Annals of Forest Research*, *55*(1), 33-42–42. https://doi.org/10.15287/afr.2012.73

Canli, K., & Çetin, B. (2014). The Moss Flora of Akdağ Mountain (Amasya, Turkey). *The Scientific World Journal*, *2014*, 860379. https://doi.org/10.1155/2014/860379

Doğan, H. H., & Öztürk, Ö. (2016). Six new *Russula* records from Turkey. *Mycotaxon*, *130*(4), 1117–1124. https://doi.org/10.5248/130.1117

DOĞAN, H., & KURT, F. (2016). New macrofungi records from Turkey and macrofungal diversity of Pozantı-Adana. *Turkish Journal of Botany*, *40*(2), 209–217. https://doi.org/10.3906/bot-1501-22

Erper, I., Turkkan, M., Karaca, G. H., & Kilic, G. (2012). New hosts for *Phyllactinia guttata* in the Black Sea Region of Turkey. *Scandinavian Journal of Forest Research*, *27*(5), 432–437. https://doi.org/10.1080/02827581.2011.649300

Eşen, D., Soysal, G., & Yildiz, O. (2015). Regenerating Eastern Beech (*Fagus orientalis* Lipsky) With Gaps of Various Sizes in the Western Black Sea Region of Turkey. D.Ü. *Ormancılık Dergisi*, *11*, 71–82.

Filipova-Marinova, M., Pavlov, D., Coolen, M., & Giosan, L. (2013). First high-resolution marinopalynological stratigraphy of Late Quaternary sediments from the central part of the Bulgarian Black Sea area. *Quaternary International*, *293*, 170–183. https://doi.org/10.1016/j.quaint.2012.05.002

Gailing, O., & Wuehlisch, G. V. (2004). Nuclear markers (AFLPs) and chloroplast microsatellites differ between *Fagus sylvatica* and *F. orientalis*. *Silvae Genetica*, *53*, 105–110. https://doi.org/10.1515/sg-2004-0019

Georgiev, G., Gjonov, I., & Sakalian, V. (2015). New Records of Longhorn Beetles (Coleoptera: Cerambycidae) in Strandzha Mountain. *Journal of the Entomological Research Society*, *17*, 73–88.

Gholizadeh, H., Naqinezhad, A., & Chytrý, M. (2019). Hyrcanian Forest Vegetation Database. *Phytocoenologia*, *49*(2)*,* 209–210. https://doi.org/10.1127/phyto/2018/0315

Goginashvili, N., & Tvauri, I. (2013). Beech forests with yew (*Fageta-taxceto*) in Georgia. International Caucasian Forestry Symposium, Artvin, Turkey, pp. 976–981.

Gömöry, D., & Paule, L. (2010). Reticulate evolution patterns in western-Eurasian beeches. *Botanica Helvetica*, *120*(1), 63–74. https://doi.org/10.1007/s00035-010-0068-y

Gülseven, O., Ayan, S., Özel, H. B., & Yer, E. N. (2019). Morphological and physiological characteristics of seedlings of different Eastern beech (*Fagus orientalis* Lipsky.) populations. *Turkish Journal of Forestry*, *20*(3), 180–186.

Jaafari, A., Najafi, A., & Zenner, E. K. (2014). Ground-based skidder traffic changes chemical soil properties in a mountainous Oriental beech (*Fagus orientalis* Lipsky) forest in Iran. *Journal of Terramechanics*, *55*, 39–46. https://doi.org/10.1016/j.jterra.2014.06.001

Japoshvili, G., Fizdale, M., Driesche, R. G. V., & Kirkitadze, G. (2015). Comparative phenology and cohort survival of beech scale (Hemiptera: Eriococcidae) in part of its Native Range (Caucasus Mountains, Georgia) and in an Invaded Area (Massachusetts, USA). *Florida Entomologist*, *98*(4), 1193–1198. https://doi.org/10.1653/024.098.0427

Köse, N., & Güner, H. T. (2012). The effect of temperature and precipitation on the intra-annual radial growth of *Fagus orientalis* Lipsky in Artvin, Turkey. *Turkish Journal of Agriculture and Forestry*, *36*(4), 501–509.

Kutbay, G., Yalçın, E., & Bilgin, A. (2003). Foliar N and P resorption and foliar nutrient concentrations in canopy and subcanopy of a *Fagus orientalis* forest. *Belgian Journal of Botany*, *136*, 35–44. https://doi.org/10.2307/20794512

Kvavadze, E. (2001). Annual modern pollen deposition in the foothills of the Lagodekhi Reservation (Caucasus, East Georgia), related to vegetation and climate. *Acta Palaeobotanica*, *41*(2): 355–364.

Martin-Benito, D., Pederson, N., Köse, N., Doğan, M., Bugmann, H., Mosulishvili, M., & Bigler, C. (2018). Pervasive effects of drought on tree growth across a wide climatic gradient in the temperate forests of the Caucasus. *Global Ecology and Biogeography*, *27*(11), 1314–1325. https://doi.org/10.1111/geb.12799

Müller, M., Lopez, P. A., Papageorgiou, A. C., Tsiripidis, I., & Gailing, O. (2019). Indications of Genetic Admixture in the Transition Zone between *Fagus sylvatica* L. and *Fagus sylvatica* ssp. Orientalis Greut. & Burd. *Diversity*, *11*(6), 90. https://doi.org/10.3390/d11060090

Oran, S., & Ozturk, S. (2010). Three lichenized fungi new to Turkey. *Mycotaxon*, *112*, 389–392. https://doi.org/10.5248/112.389

Ören, M., Uyar, G., & Keçeli̇, T. (2012). The bryophyte flora of the western part of the Küre Mountains (Bartın, Kastamonu), Turkey. *Turkish Journal of Botany*, *36*(5), 538-557. https://doi.org/10.3906/bot-1111-2

Özbayram, A. K., & Kulaç, Ş. (2018). Bir Doğu Kayını Meşceresinde Farklı Sosyal Sınıftaki Ağaçlarda Günlük Ekofizyolojik Tepkiler. *Kahramanmaraş Sütçü İmam Üniversitesi Tarım ve Doğa Dergisi*, *21*(3), 297–303. https://doi.org/10.18016/ksudobil.311776

Öztürk, Ş. Y., & Güvenç, Ş. (2010). Comparison of the epiphytic lichen communities growing on various tree species on Mt. Uludağ (Bursa, Turkey). *Turkish Journal of Botany*, *34*, 449-456 <https://doi.org/10.3906/bot-0905-12>

Paffetti, D., Vettori, C., Caramelli, D., Vernesi, C., Lari, M., Paganelli, A., Paule, L., & Giannini, R. (2007). Unexpected presence of *Fagus orientalis* complex in Italy as inferred from 45,000-year-old DNA pollen samples from Venice lagoon. *BMC Evolutionary Biology*, *7,* S6. https://doi.org/10.1186/1471-2148-7-S2-S6

Panahi, P., Jamzad, Z., Pourhashemi, M., & Hasaninejad, M. (2017). Morphological variation of *Fagus orientalis* Lipsky in the Hyrcanian forests of Iran. *The Iranian Journal of Botany*, *23*(1), 37–47. https://doi.org/10.22092/ijb.2017.111920

Peck, J. E., & Zenner, E. K. (2019). Common ground among beech forest development stages: Matrix versus stage-typical live tree structure. *Journal of Vegetation Science*, *30*(5), 893–904. https://doi.org/10.1111/jvs.12791

Salehi Shanjani, P. (2011). Differences in genetic structure among *Fagus orientalis* Lipsky (Oriental Beech) populations under different management conditions: implications for in situ gene conservation. *Journal of Sciences, Islamic Republic of Iran*, *22*(1), 5–17.

Salehi Shanjani, P., Vendramin, G. G., & Calagari, M. (2010). Genetic diversity and differentiation of *Fagus orientalis* Lipsky in Hyrcanian forests revealed by nuclear and chloroplast microsatellite markers. *Conservation Genetics*, *11*(6), 2321–2331. https://doi.org/10.1007/s10592-010-0118-4

Topaçoğlu, O., & Genç, E. (2019). Forest Edge Effects on Seedlings in Mixed Oriental Beech (*Fagus orientalis* Lipsky) - Scots Pine (*Pinus sylvestris* L.) Stands. *Applied Ecology and Environmental Research*, *17*(2), 2219-2231. http://dx.doi.org/10.15666/aeer/1702_22192231

Uzun, Y., & Kaya, A. (2019). *Elaphomyces granulatus*, a new hypogeous Ascomycete record for Turkey. *Kahramanmaraş Sütçü İmam Üniversitesi Tarım ve Doğa Dergisi*, *22*, 85–88.

Vacek, Z., Vacek, S., Eşen, D., Yildiz, O., Král, J., & Gallo, J. (2020). Effect of Invasive *Rhododendron ponticum* L. on Natural Regeneration and Structure of *Fagus orientalis* Lipsky Forests in the Black Sea Region. *Forests*, *11*(5), 603. https://doi.org/10.3390/f11050603

Vettori, C., Paffetti, D., Paule, L., & Giannini, R. (2004). Identification of the *Fagus sylvatica* L. and *Fagus orientalis* Lipsky species and intraspecific variability. *Forest Genetics*, *10*, 223–230.

Yaman, B., Köse, N., Özel, H. B., & Şahan, E. A. (2020). The effect of climate on the radial growth of Oriental Beech in the Southern limit of its distribution area. *Forestist*, *70*(1), 53–59. https://doi.org/10.5152/forestist.2020.19024

Yazici, K., Aptroot, A., & Aslan, A. (2007). Lichen biota of Zonguldak, Turkey. *Mycotaxon*, *102*, 257-260.

Yilmaz, M. (2008). Three-year storage of oriental beechnuts (*Fagus orientalis* Lipsky). *European Journal of Forest Research*, *127*(5), 441. https://doi.org/10.1007/s10342-008-0227-5

Yilmaz, O. Y., & Yilmaz, H. (2020). Can shelterwood logging maintain herb layer diversity in a beech forest in Turkey? *Applied Ecology and Environmental Research*, *18*, 487–498. https://doi.org/10.15666/aeer/1801_487498

Yücesan, Z., Özçelik, S., & Oktan, E. (2015). Effects of thinning on stand structure and tree stability in an afforested Oriental beech (*Fagus orientalis* Lipsky) stand in northeast Turkey. *Journal of Forestry Research*, *26*(1), 123–129. https://doi.org/10.1007/s11676-015-0028-x

# Appendix S3: Results


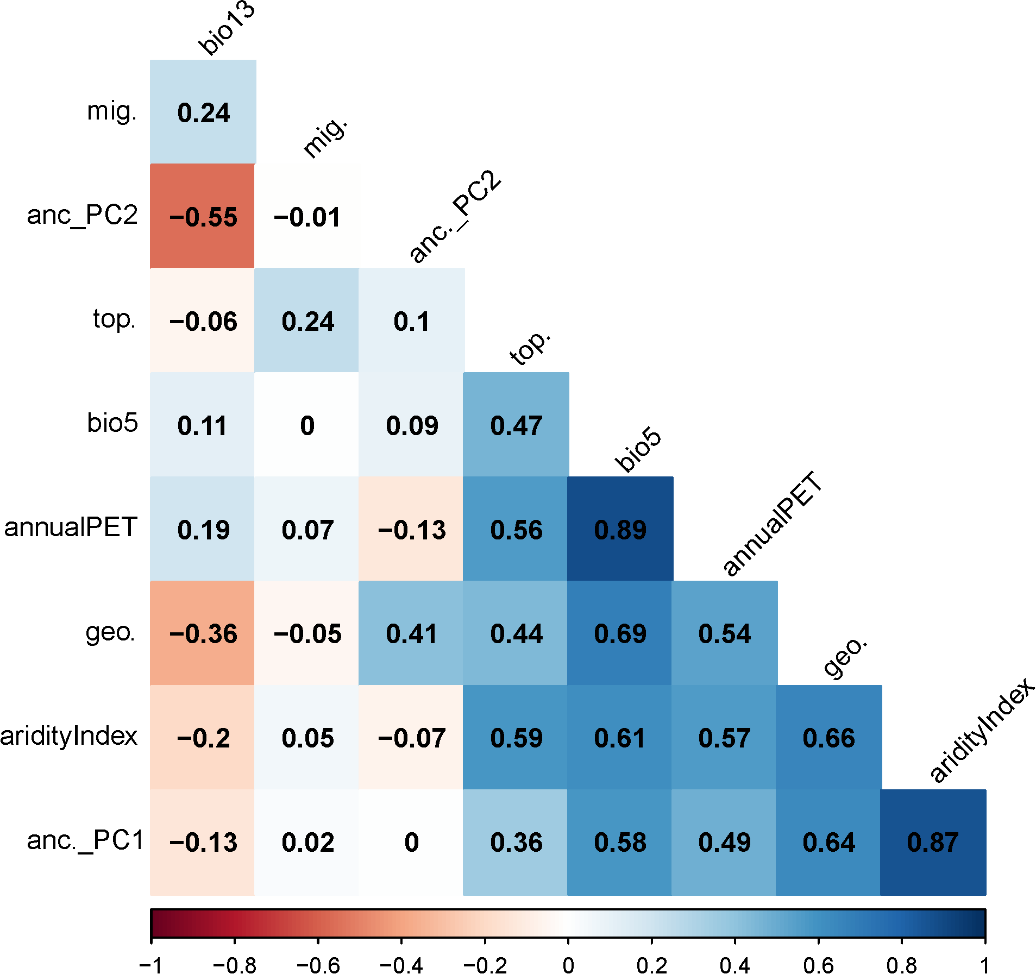


**Figure S3.2** The correlation matrix among explanatory variables used in the series of distance-based redundancy analyses (dbRDA), including climate (*clim*., the maximum temperature ‘bio 5’, precipitation of wettest month ‘bio13’, aridity index ‘aridityIndex’ and annual potential evapotranspiration ‘annualPET’), geography (*geo*.), recent migration (*mig*.), the first two PCs of genetic structure (*anc*._PC1, *anc*._PC2) and topographic heterogeneity (*top*.)

**Table S3.2** Characterization and polymorphism of nuclear microsatellite loci (nSSRs) originally developed for *Fagus sylvatica* (Pastorelli et al., 2003; Pluess & Määttänen, 2013) used in this study

| **Locus** | **Primer sequences** | **Repeated motif** | **Allele size range (bp)** | **5' Dye:** | **A** | **H_O_** | **H_E_** | **F_IS_** | **Null** |
| --- | --- | --- | --- | --- | --- | --- | --- | --- | --- |
| FS1-15 | TCAAACCCAGTAAATTTCTCA | (GA)_26_ | 83-133 | VIC | 34 | 0.722 | 0.938 | 0.23 | 0.13 |
|  | GCCTCAATGAACTCAAAAAC |  |  |  |  |  |  |  |  |
| FS1-03 | CACAGCTTGACACATTCCAAC | (GA)_18_ | 86-112 | PET | 15 | 0.294 | 0.317 | 0.072 | 0.02 |
|  | TGGTAAAGCACTTTTTCCCACT |  |  |  |  |  |  |  |  |
| FS3-04 | AGATGCACCACTTCAAATTC | (GCT)_5_(GTT)_3_(GCT)_23_ | 192-204 | NED | 15 | 0.225 | 0.241 | 0.063 | 0.03 |
|  | TCTCCTCAGCAACATACCTC |  |  |  |  |  |  |  |  |
| FS4-46 | GCAGTCCTCCACCATTACTA | (TGA)_23_ | 176-272 | 6-FAM | 56 | 0.462 | 0.797 | 0.42 | 0.22 |
|  | TACAACAGCAGGCTATCCAT |  |  |  |  |  |  |  |  |
| FCM5 | ACTGGGACAAAAAAACAAAA | (AG)_10_ | 272-338 | VIC | 34 | 0.485 | 0.724 | 0.33 | 0.15 |
|  | GAAGGACCAAGGCACATAAA |  |  |  |  |  |  |  |  |
| Fagsyl_001018 | AATATCAGGGAGGCAGCACC | (CA) | 107–126 | VIC | 16 | 0.827 | 0.826 | -0.002 | 0.01 |
|  | CGAGATGGACTTCTAAGTTTTATTTGC |  |  |  |  |  |  |  |  |
| Fagsyl_001217 | GGATGGGTTTTTGGCTCAGG | (CA) | 133–156 | NED | 17 | 0.545 | 0.689 | 0.208 | 0.09 |
|  | CGTTGTCATGCAGGAGTGTG |  |  |  |  |  |  |  |  |
| Fagsyl_003994 | ACAAAGGAATCGTGGAGCTG | (GA) | 110–136 | PET | 22 | 0.811 | 0.834 | 0.028 | 0.02 |
|  | ACACATTCTGCCTCAAAGTACC |  |  |  |  |  |  |  |  |
| Fagsyl_003093 | TCATCACCGAGACAAGGGAC | (CA) | 148–205 | PET | 18 | 0.755 | 0.815 | 0.073 | 0.03 |
|  | ATGGTGGTGTGGAAGCTAGG |  |  |  |  |  |  |  |  |
| Fagsyl_003849 | GCTTCGTTCTTCGGCATCTC | (CT) | 98–128 | NED | 17 | 0.849 | 0.886 | 0.042 | 0.02 |
|  | AATAGCACAAATAGCCCGCC |  |  |  |  |  |  |  |  |
| Fagsyl_007038 | ACCAGCAAATCCACGTATTAAC | (TG) | 152–166 | 6-FAM | 22 | 0.714 | 0.75 | 0.047 | 0.02 |
|  | GTGGGCTTGTGACTTGCTTC |  |  |  |  |  |  |  |  |
| Fagsyl_000909 | TCACTTGATGCCACATGCAC | (AC) | 150–166 | VIC | 14 | 0.606 | 0.631 | 0.039 | 0.02 |
|  | CGCCCATCGCTTGAAGTAAG |  |  |  |  |  |  |  |  |
| Fagsyl_006075 | TGGATGCAATCCGTGAACTG | (TG) | 156–269 | NED | 13 | 0.766 | 0.803 | 0.046 | 0.02 |
|  | TGGTCTCTTCCTCGTCCAAG |  |  |  |  |  |  |  |  |
| **Average** | | | | | **22.5** | **0.620** | **0.712** | **0.123** | **0.059** |

A – number of alleles; H_O_ – observed heterozygotes; H_E_ – expected heterozygotes; F_IS_ – inbreeding coefficient; Null – null allele frequency

**Table S3.3** The pairwise differentiation (F_ST_) among studied populations of oriental beech in the South Caucasus computed in FreeNA with the Excluding Null Alleles (ENA) correction. The significance was assessed based on 9999 permutations. All values are in the range of 95% CI. The colour gradient represents different F_ST_ values, from low (dark blue) to high (dark red) genetic differentiation:

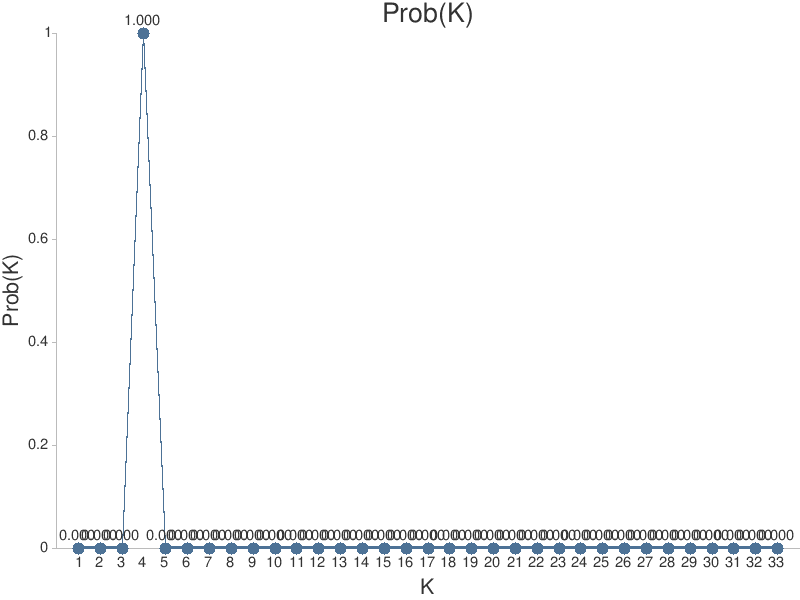


**Figure S3.3** The optimal K number of genetic clusters according to the log probability of the data (Ln Pr (X|K) (Pritchard et al., 2000)

# References

Pastorelli, R., Smulders, M. J. M., Westende, W. P. C. V., Vosman, B., Giannini, R., Vettori, C., & Vendramin, G. G. (2003). Characterization of microsatellite markers in *Fagus sylvatica* L. and *Fagus orientalis* Lipsky. *Molecular Ecology Notes*, *3*(1), 76–78. https://doi.org/10.1046/j.1471-8286.2003.00355.x

Pluess, A. R., & Määttänen, K. (2013). Characterization of eighteen novel microsatellite markers and multiplex PCR protocol for Fagus sylvatica. *Conservation Genetics Resources*, *5*(2), 311–314. https://doi.org/10.1007/s12686-012-9791-6

QGIS.org. (2022). *QGIS Geographic Information System. QGIS Association. Http://www.qgis.org*.
